# Anankastia or Psychoticism? Which One Is Better Suited for the Fifth Trait in the Pathological Big Five: Insight From the Circumplex of Personality Metatraits Perspective

**DOI:** 10.3389/fpsyt.2021.648386

**Published:** 2021-10-14

**Authors:** Włodzimierz Strus, Patryk Łakuta, Jan Cieciuch

**Affiliations:** ^1^Institute of Psychology, Cardinal Stefan Wyszyński University in Warsaw, Warsaw, Poland; ^2^University Research Priority Program Social Networks, University of Zurich, Zurich, Switzerland

**Keywords:** ICD-11, DSM-5, Big Five, Circumplex of Personality Metatraits, personality disorders

## Abstract

Both the ICD-11 and the DSM-5 (Section III) classification systems introduced dimensional models of personality disorders, with five broad domains called *the Pathological Big Five*. Nevertheless, despite large congruence between the two models, there are also substantial differences between them, with the most evident being the conceptualization of the fifth dimension: Anankastia in the ICD-11 vs. Psychoticism in the DSM-5. The current paper seeks an answer to the question of which domain is structurally better justified as the fifth trait in the dimensional model of personality disorders. For this purpose, we provided both a conceptual and empirical comparison of the ICD-11 and the DSM-5 models, adopting the Circumplex of Personality Metatraits—a comprehensive model of personality structure built on the basis of the higher-order factors of the Big Five—as a reference framework. Two studies were conducted: the first on a sample of 242 adults (52.9% female; *M*_age_ = 30.63, *SD*_age_ = 11.82 years), and the second on a sample of 355 adults (50.1% female; *M*_age_ = 29.97, *SD*_age_ = 12.26 years) from the non-clinical population. The Personality Inventory for ICD-11 (PiCD), the Personality Inventory for DSM-5 (PID-5), and the Circumplex of Personality Metatraits Questionnaire–Short Form (CPM-Q-SF) were administered in both studies, together with the PID-5BF+M algorithm for measuring a common (ICD-11 + DSM-5) six-domain model. Obtained empirical findings generally support our conceptual considerations that the ICD-11 model more comprehensively covered the area of personality pathology than the DSM-5 model, with Anankastia revealed as a more specific domain of personality disorders as well as more cohesively located within the overall personality structure, in comparison to Psychoticism. Moreover, the results corroborated the bipolar relations of Anankastia vs. Disinhibition domains. These results also correspond with the pattern of relationships found in reference to the Big Five domains of normal personality, which were also included in the current research. All our findings were discussed in the context of suggestions for the content and conceptualization of pathological personality traits that flow from the CPM as a comprehensive model of personality structure including both pathological and normal poles of personality dimensions.

## Introduction

Shifting from categorical models toward a dimensional approach to personality disorders represents a historically significant step toward building an empirically driven (and theoretically justified) diagnostic system. That transformation can be seen in both public-health-focused authoritative classification systems—the fifth edition of the American Psychiatric Association's (APA) Diagnostic and Statistical Manual of Mental Disorders [DSM-5; (1)] with the Alternative Model of Personality Disorders (AMPD) and the 11th edition of the International Classification of Diseases (ICD-11) proposed by the World Health Organization (2). Each in its own way has made modifications to acknowledge the usefulness of dimensional features that are relevant to personality disorders and to incorporate these dimensional features into their diagnostic systems, endeavoring to improve their validity and clinical utility. Dimensionality can be argued for all mental disorders (at large), but for personality disorders, the case has a compelling rationale (3–7).

For describing personality disorders, both DSM-5 AMPD and ICD-11 propose five pathological dimensions that are related to the Big Five personality traits treated as a model of normal personality (also known as the Five-Factor Model—FFM for short). In that way, both dysfunctional models lean on a model that is extensively validated within research in the field of personality and individual differences (8). However, they differ in one point: DSM-5 AMPD distinguishes five traits that are maladaptive versions of the normal Big Five/FFM while ICD-11 includes Anankastia instead of Psychoticism, having two domains related to Conscientiousness and none related to Openness. In our paper, we seek to answer which pathological Big Five is more justified from the point of view of recent advances in research on personality structure, and especially from the Circumplex of Personality Metatraits [CPM; (9, 10)] that has been developed as the most comprehensive model in the personality traits research tradition.

### Two Pathological Big Fives: Similarities and Differences Between ICD-11 and DSM-5 Dimensional Models of Personality Disorders

The AMPD was incorporated into Section III of DSM-5 for “Emerging Measures and Models” in need of further study (1), to offer a hybrid model for the diagnosis of personality disorders (PDs). Of crucial importance in the DSM-5 AMPD (hereafter called briefly the DSM-5) are two components: (1) Criterion A that refers to the overall level of personality functioning and (2) Criterion B that is constituted by the five-dimensional model of personality pathology. Moreover, both Criterion A and Criterion B are formulated together with recreated sets of criteria devised for six diagnostic categories of specific PDs. Similarly, to the DSM-5, the ICD-11 model of PDs, along with a rating of overall severity of personality dysfunction includes a five-domain dimensional model to provide an opportunity to point to where the focus of the disorder is manifested (2). An important point to recognize is that the ICD-11 has avoided a hybrid approach by not using any nosological entities (specific categories of PDs) and having a single dimension of severity for all personality dysfunctions, ranging from non-disordered personality at one end to severe personality disorder at the other (2, 11). The potential clinical utility of both multidimensional personality trait models lies in their ability to focus attention on multiple relevant areas of personality in each individual patient rather than focusing attention on the identification of a diagnostic label.

**Figure 1:**
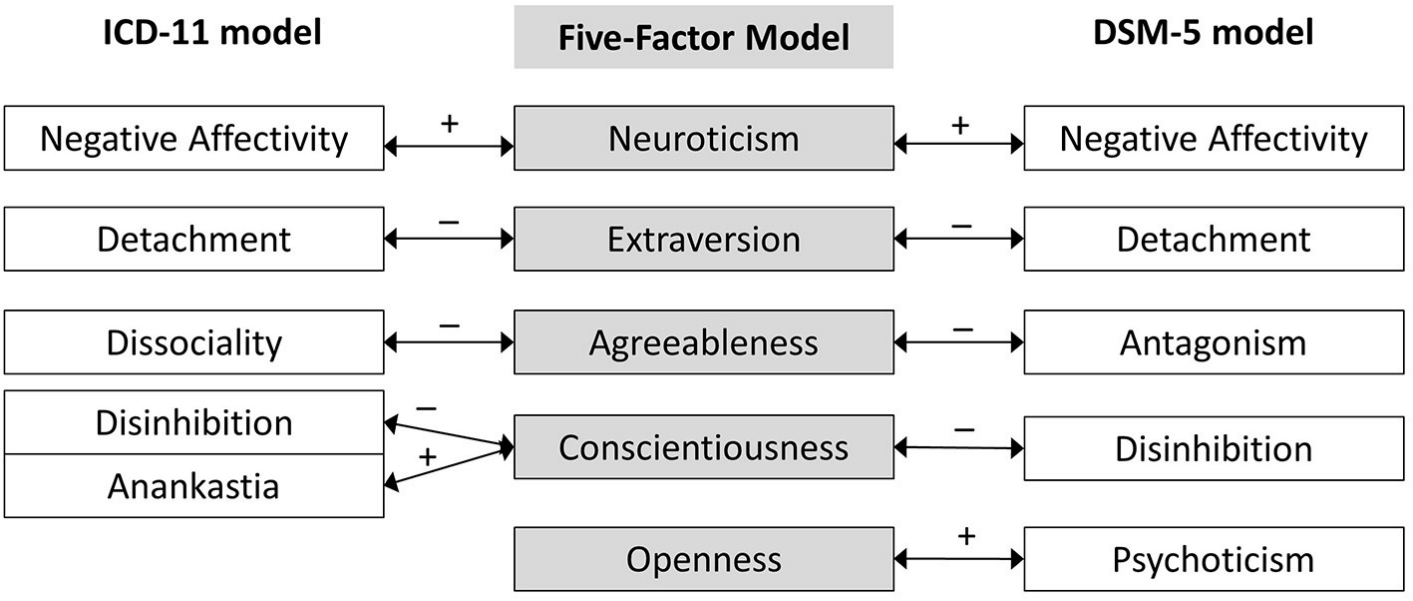
Juxtaposition of the ICD-11 and DSM-5 models of pathological Big Five domains with the FFM model of normal personality structure.

A significant origin, inspiration, and context of both DSM-5 and ICD-11 models of pathological traits is the FFM model of normal personality. Namely, pathological dimensions of DSM-5 and ICD-11 models are supposed to be the maladaptive extremes of the FFM normal personality dimensions. The DSM-5 trait model involves all five FFM domains (see Figure 1), namely Neuroticism, Introversion (opposite pole of Extraversion), low Conscientiousness, Antagonism (negative pole of Agreeableness) and Openness to Experience (for a review, see (12)]. Having incorporated these five prominent trait domains, the DSM-5 model links the broad literature of normal personality structure.

The same point largely applies to the ICD-11 trait model, however, with one important exception. The domain traits in the ICD-11 model have been carefully distilled from broad empirical evidence on personality disorders [for a more detailed overview, see (11)], and four of the traits are essentially the same as four of those identified by the DSM-5. The domains incorporated within the ICD-11 are likewise aligned with the FFM: Negative Affectivity with Neuroticism, Detachment with low Extraversion, Dissociality with low Agreeableness, Disinhibition with low Conscientiousness, and Anankastia with high Conscientiousness (13). Essentially, the ICD-11 trait structure can be seen then as a maladaptive variant of the FFM model of normal personality, except Openness to Experience that is not represented and, in some sense, also Conscientiousness that is represented in both of its extreme (maladaptive) poles by Disinhibition and Anankastia (see Figure 1).

Therefore, both models have five broad trait domains, but share only four, i.e., Negative Affectivity, Detachment, Disinhibition, and a fourth called Antagonism (in DSM-5) or Dissociality (in ICD-11). So, each model poses a fifth trait-domain that the other does not. The ICD-11 trait model does not include the Psychoticism domain, concerning proneness to eccentric or delusional behaviors, cognitions, and thought processes. In turn, the DSM-5 trait model does not include the domain of Anankastia, concerning perfectionism, rigidly sticking to the norm and obligations or emotional and behavioral constraint (e.g., inflexible control and perseveration). Psychoticism was omitted by ICD-11 not because it was seen as conceptually irrelevant, but rather, because psychotic phenomena are described in a separate part of the ICD. It is a part of a long-established history that WHO does not consider schizotypal to be a personality disorder phenomenon, and, as such, in the ICD it is classified as part of the schizophrenia spectrum disorders. Anankastia was omitted by DSM-5, although the closely related Compulsivity domain was included within the original DSM-5 proposal (14). However, in the end, this domain was deleted in favor of parsimony (15), and only two of the original trait-facets from this domain were preserved in the final version of the DSM-5 model [i.e., rigid perfectionism and perseveration within Disinhibition and Negative Affectivity domains, respectively; (1)]. Taking into account that the obsessive-compulsive phenomenon is of crucial clinical importance due to it being the most common PD (16), these characteristics could be deemed underrepresented in the DSM-5 model, and, in general, the lack of Anankastia in the Pathological Big Five can be seen as neglecting an important domain.

Including Anankastia seems to be clinically justified and supported by analyses of this domain structure using the new ICD-11 model that produces strong support for good discrimination and validity of Anankastia across different cultural groups [discussed in more detail in (11)]. On the other hand, however, it breaks the elegant parallel to the normal FFM and the unipolar nature of dimensions differentiated in the pathological Big Five. Anankastia vs. Disinhibition seems to form a sort of one bipolarly defined dimension (at one pole by Anankastia and at the other pole by Disinhibition), which has been supported in many studies (17–20); see also (7)]. Therefore, one can say that the ICD-11 model is a model with five pathological domains, but only four pathological factors, making the bridge to the normal FFM less obvious.

Summing up, it seems that the DSM-5 model better fits the FFM of normal personality structure than the ICD-11 model (see Figure 1). However, on the other hand, the elegant Big Five structure of DSM-5 turned out to face some problems, including medium to large intercorrelations within the traits and problematic discriminant validity, particularly of the Psychoticism dimension (20–27). As noted by McCabe and Widiger (20), one compelling explanation can be that there is a general factor of personality disorder that saturates all measures of PD (15). However, if this is the reason for the problematic discriminant validity of the DSM-5 model then it would have also had a comparable impact on the ICD-11 dimensions, but as shown that was not the case (20). Thus, the status of Psychoticism within the Pathological Big Five remains vague. From a conceptual point of view, Psychoticism is expected to map specifically onto the FFM Openness domain (in particular onto some facets, e.g., fantasy, imagination), nonetheless many studies have shown that Psychoticism has unexpected weak relations to Openness, much weaker than its links to Agreeableness, Conscientiousness, Neuroticism, and to other DSM-5 traits [e.g., (20, 27)]. Of note is also that the metanalytical evidence (28) has shown that all three of the trait-facets from the Psychoticism domain (i.e., eccentricity, perceptual dysregulation, and unusual beliefs) reached meaningful associations with antisocial PD, although they are not part of the proposed criterion profile for this disorder (1). These findings are generally congruent with Eysenck's proposal (29) that disinhibition and psychosis fall on the same continuum, with the former indicating a progression toward the latter. A complementary concern is that the overall pattern of complex associations of Psychoticism could be in part due to its heterogeneity or non-specificity with respect to other traits in the model.

As we can see, there is a disagreement among different authority systems of PD diagnosis regarding the issue of Psychoticism or Anankastia. One can say that the simplest solution to this problem is not asking which one is better or more justified, but to integrate both Anankastia and Psychoticism within the joint six-domain model. Some such interesting efforts to harmonize the two PD systems and combined ICD-11 and DSM-5 trait domains into one model have indeed been recently made (30–32). However, on the other hand, eclecticism and integration are not always a good answer. Moreover, given that the Psychoticism vs. Anankastia problem reflects old APA vs. WHO controversy relating to the core of personality disorders, it seems to be worth asking (and search for the answers) which model of pathological dimensions—DSM-5's or ICD-11's—is more justified. We have made an attempt to answer this question from the perspective of the current state of the art in the knowledge of the structure of personality.

It is worth noting that the problematic discriminant validity obtained for the PID-5 assessment of the DSM-5 traits can be seen in some way as the FFM's heritage. Actually, the Pathological Big Five models—particularly in the DSM-5 version—suffer from similar problems as the FFM model of personality structure does (such as evidence challenging the orthogonality of five basic dimensions). However, the past 20 years have seen the rise of a few serious alternatives to the predominant FFM that aimed to solve these problems. In this paper, we offer a change in the point of view and look at models of pathological personality dimensions from the perspective of recent advances in theory and research on personality structure.

### Recent Advances in Personality Structure and the Circumplex of Personality Metatraits

The Big Five/FFM has recently been criticized for claims that the five personality dimensions are the very basic ones. There are two main lines of the critique: The first one challenges the number and proposes a sixth dimension [Honesty-Humility in HEXACO or Big Six model;(33)] while the second one challenges the orthogonality of basic dimensions and proposes two basic higher-order factors above the Big Five (34–36). Ashton and Lee (33) made a strong case for the Big Six/HEXACO model, demonstrating some advantages over the Big Five/FFM, and a better theoretical interpretation of personality variation (37–39). However, the pathological trait dimensions of the ICD-11 and DSM-5 are less congruent with the personality space of the Big Six/HEXACO than the Big Five/FFM. There is also no similarity between HEXACO and the joint (ICD-11 plus DSM-5) six-domain model of pathological traits (32) besides just the number of traits (six). In contrast, the second line of the criticism against the Big Five/FFM seems to be much more promising for the conceptualization of the pathological trait-domains. Digman (34) as well as DeYoung et al. (35) describe the highest level of personality structure as being a compound of two very broad superfactors (called metatraits) located above the Big Five: Alpha/Stability (constituted by Agreeableness, Conscientiousness, and Emotional Stability) and Beta/Plasticity [comprised of Extraversion and Intellect/Openness; see (36)]. This Two-Factor Model of personality corresponds reversely to two broad classes (or factors) of psychopathology, namely externalizing and internalizing problems (40, 41). In turn, on the basis of this model, Strus et al. (42) have developed the Circumplex of Personality Metatraits (CPM), which has already manifested a strong integrative potential (9, 36, 43) and seems to be worth trying to use in the conceptualization of the pathological traits.

**Figure 2:**
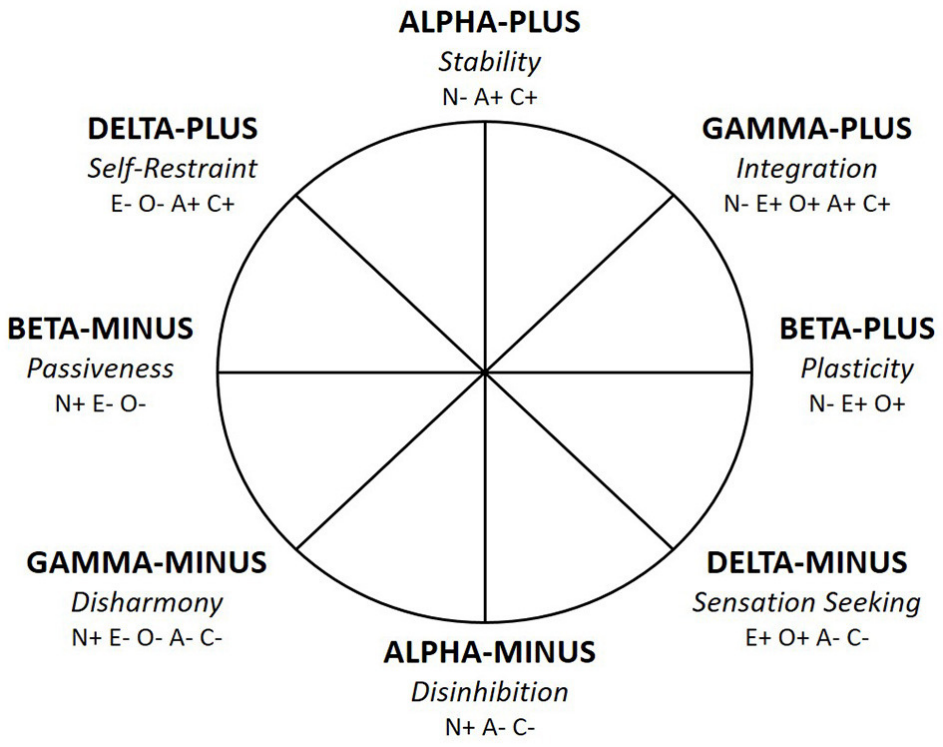
The Circumplex of Personality Metatraits—revised (10). N, Neuroticism; E, Extraversion; O, Openness to Experience; A, Agreeableness; C, Conscientiousness; + denotes the positive pole of a trait; – denotes the negative pole of a trait.

Within the CPM model, Alpha/Stability and Beta/Plasticity are treated as orthogonal dimensions of the circumplex space and are complemented by two other metatraits (see Figure 2). The first of them is Gamma/Integration being a reflection of the General Factor of Personality (GFP), which it is no longer proposed to be located at the top of the hierarchy of personality traits but at the same level as Alpha/Stability and Beta/Plasticity in the circumplex structure. Gamma/Integration encompasses the most functional vs. most maladaptive configuration of personality traits. In turn, the last, fourth dimension fully complements the conceptual space of the circumplex—Delta/Self-Restraint is derived from high/low Stability vs. low/high Plasticity, broadly reflecting findings of research on personality structure [for an overview, see (9, 10, 43)].

The CPM is a holistic model of personality, combining the dynamic between health and pathology. It is worth noting in this context that recent refinement of the model brings relocation of Neuroticism from Alpha to Gamma (10). As such, the CPM clearly encompasses three main aspects of the Neuroticism domain: anxiety-based core features located centrally within Gamma-Minus; externalizing-hostile features located within Alpha-Minus; and an internalizing-self-conscious-dependent (or submissive) features within Beta-Minus. This relocation of Neuroticism enhanced the meaning of Gamma (44) as the metatrait reflecting the level of well-being and mental-health vs. psychopathology, with Gamma-Minus/Disharmony being the counterpart of the general factors of psychopathology (*p* factor) and general factor of personality disorder [g-PD; (45, 46)]. In turn, Delta (now practically unrelated to Neuroticism or related to its medium level) became the metatrait that delineates a boundary between healthy and problem prone personality, as well as determines the type of psychological problems when the low intensity of Gamma is the case. A full description of each pole of four metatraits is provided in Table 1 (see also Figure 2).

**Table 1:** Description of the eight metatraits in the Circumplex of Personality Metatraits revised.

**Metatrait**	**Big five configuration**	**Meaning**
Delta-Plus (Self-Restraint)	E−, O−, A+, C+ (N_0_)	Low emotionality (both negative and positive), high behavioral and emotional control, meticulousness, and perfectionistic tendencies as well as modesty, conventionality, and severe social adjustment.
Alpha-Plus (Stability)	N−, A+, C+ (E_0_, O_0_)	Stability in the area of emotional, motivational, and social functioning, expressed as a general social adaptation tendency, an ethical attitude toward the world, benevolence, and calmness, as well as the ability to delay gratification, diligence and perseverance.
Gamma-Plus (Integration)	N−, E+, O+, A+, C+	Well-being, a warm and prosocial attitude toward people, both intra- and interpersonal balance and harmony; serenity, openness to the world in all its richness, as well as endurance and effectiveness in attaining important goals.
Beta-Plus (Plasticity)	N−, E+, O+ (A_0_, C_0_)	Cognitive and behavioral openness to change and engagement to new experiences, a tendency to explore, self-confidence, initiative and invention in social relations, as well as enthusiasm, and an orientation toward personal growth.
Delta-Minus (Sensation-Seeking)	E+, O+, A−, C− (N_0_)	Broadly defined impulsiveness, recklessness, emotional volatility, stimulation seeking and risk taking; self-enhancement and hedonistic tendencies as well as interpersonal dominance and expansiveness.
Alpha-Minus (Disinhibition)	N+, A−, C− (E_0_, O_0_)	High level of antisocial tendencies underpinned by unsustainability, low frustration tolerance, and egotism, as well as aggression and antagonism toward people, social norms, and obligations.
Gamma-Minus (Disharmony)	N+, E−, O−, A−, C−	Inaccessibility, coldness and distrust in interpersonal relations; negative affectivity and low self-worthiness; depressiveness, pessimism, and proneness to suffer from psychological problems.
Beta-Minus (Passiveness)	N+, E−, O− (A_0_, C_0_)	Social avoidance and timidity, together with submissiveness and dependency in close relationships; cognitive and behavioral passivity and inhibition; some type of stagnation, apathy, and tendency for anhedonia.

*For abbreviations see Figure 2; 0 denotes a medium level of trait intensity; + denotes a high level of trait intensity; – denotes a low level of trait intensity. Adapted and modified from Strus and Cieciuch (9, 10)*.

The key advantage of the CPM is that it serves as a matrix for a comprehensive, wide-ranging theoretical integration and precise testing of other models. The CPM has amassed a considerable body of empirical support, including testing a broad set of constructs described by various concepts or theories of personality, emotion and motivation, mental health, and psychopathology (10, 47–50). Changing the perspective of analysis from hierarchical to circumplexical provides such a clarification framework for structural testing of constructs from the same level of personality organization and specifying their mutual relationships. Therefore, the CPM broad conceptual framework along with its circumplex rather than hierarchical structure enables the main pathological traits to be identified and their mutual relationships to be precisely described.

### The Current Study: The Circumplex of Personality Metatraits as a Reference Frame for the Pathological Big Five Models

Building upon the CPM structure, in the present study we look at the possibility to capture the dimensional structure of both PD models in a broader conceptual perspective, especially to clarify and systematize their mutual relationships, and, therefore to enrich the discussion on potential venues for their integration or choice between them. The circumplex matrix of the CPM, which is claimed to capture very basic dimensions of personality, provides the opportunity for a thorough comparison of two catalogs of pathological personality trait domains derived from the ICD-11 and DSM−5 models. The reference frame offered by CPM especially helps to assess how comprehensively each of the two competitive Pathological Big Five models conceptualizes and captures the maladaptive side of personality.

In particular, changing the reference frames from the FFM to the CPM allows us to address the following problems: (1) intercorrelations between pathological personality dimensions can be precisely theoretically predicted due to the circumplex nature of the CPM; (2) the structural comprehensiveness of a model that differentiates some dimensions to describe a given phenomenon (in this case personality disorders) can be assessed, as the CPM can help to identify some “empty spaces” in the structure that are not captured by any dimensions differentiated in a given model; (3) redundancy or overlapping of some dimensions or, in other words, concerns with respect to discriminant validity of the dimensions distinguished in a given model can be examined. If some constructs could not be theoretically located in one specific place of the CPM model (or there are two constructs placed in the same location), there is a risk that they share their content with several dimensions, and, as such should be reconceptualized. Notably, being a reflection of the integrative potential of the CPM, correlations between traits are allowed—indeed, they are even precisely determined—providing a direct test of connections between all trait domains.

Given its circumplex structure, the CPM as a broad model of personality structure creates a system of coordinates integrating various constructs. Specifically, it enables empirical testing of hypotheses regarding precisely formulated angles and coordinates of other variables. In the current research, we delineated theoretical locations of two Pathological Big Fives models, i.e., (A) the ICD-11 model and (B) the DSM-5 model. Then, we tested our predictions examining the congruence between theoretical expectations and empirical locations of the pathological personality traits in the CPM framework additionally including (C) the conjoint six-domain model.

**Figure 3:**
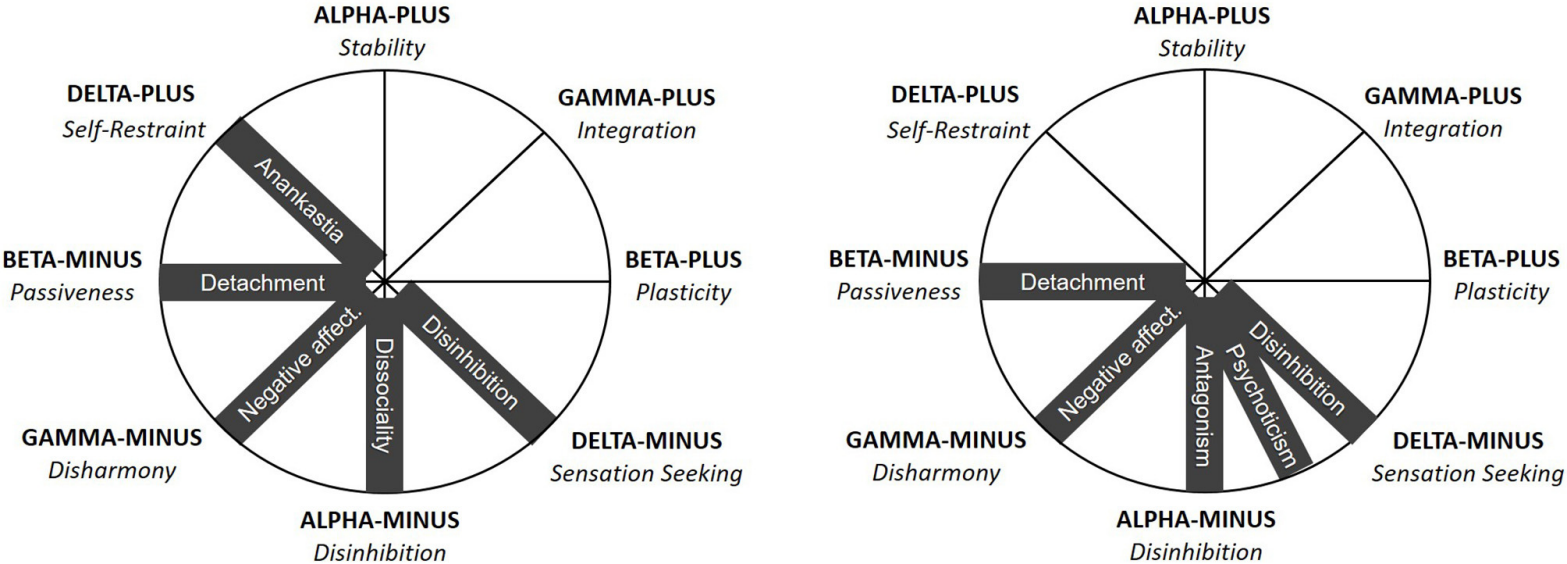
Location of the pathological traits from the ICD-11 (on the left) and DSM-5 (on the right) models within the Circumplex of Personality Metatraits. Pathological traits are depicted on black background. The location of the Disinhibition domain in both ICD-11 and DSM-5 models within Delta-Minus/Sensation Seeking rather than Alpha-Minus/Disinhibition is justified by the meaning of these dimensions.

Figure 3 presents the theoretical location of pathological traits distinguished in the DSM-5 and ICD-11 model within the CPM. Regarding four shared by the DSM-5 and ICD-11 domains, their locations within the CPM model are analogous. Negative Affectivity domains (from both DSM-5 and ICD-11) are expected (H1) to be most strongly related to Gamma-minus, as all of them include a tendency to experience negative emotions, low self-esteem, or distrust (1, 2). Detachment (from both DSM-5 and ICD-11) is predicted (H2) to be most strongly related to Beta-Minus, as all these dimensions contain social avoidance, emotional indifference, or a tendency for anhedonia (1, 2). In turn, Antagonism (in DSM-5) and Dissociality (in ICD-11) are predicted (H3) to be located in Alpha-Minus, as all of them include antisocial tendencies, aggressiveness, and self-centeredness (1, 2). Finally, Disinhibition (from both DSM-5 and ICD-11) is supposed to (H4) be placed in Delta-Minus, as these dimensions share common features of impulsivity, recklessness, and volatility [or risk taking; (1, 2)]. The latter location seems to be justified in terms of psychological meaning, despite the fact that the “disinhibition” label in the CPM model has been assigned to Alpha-Minus rather than Delta-minus (see Figures 2, 3).

However, the most interesting are the location of the last, fifth dimensions of both pathological trait models. Regarding the DSM-5 model, the location of the Psychoticism domain within the CPM space seems to be ambiguous. On one hand, it is supposed to be the maladaptive variant of high Openness to Experience and one of its facets concerns eccentric behaviors, which suggests that the location of the Psychoticism domain is between Beta-Plus and Delta-Minus. On the other hand, Psychoticism is a pathological trait, which is crucial for schizotypal disorders and therefore related to Detachment/Introversion. This, together with the revealed pattern of correlations between Psychoticism and other DSM-5, as well as FFM, traits suggest its location somewhere below the Delta-Minus dimension. Considering also Eysenck's (29) proposal—relating Psychoticism with antisocial tendencies—we finally hypothesized Psychoticism (H5) as being located between Delta-Minus and Alpha-Minus. However, this location seems to be less specific or justified, which corresponds with greater heterogeneity of Psychoticism with respect to other dimensions in the DSM-5 model and suggests that Psychoticism—as concerning mainly cognitive dysregulations—could adhere to the cognitive or intellectual-ability area of functioning rather than to (maladaptive) personality traits. For example, having references to delusions [e.g., thought-control experiences; (1)] is particularly problematic, because delusions are not typically understood to be (related to) personality traits (51). In contrast, Anankastia from the ICD-11 model seems to be cohesively related (H6) to Delta-Plus, as both dimensions include such characteristics as perfectionistic tendencies, concern with following rules and norms as well as high emotional and behavioral control (2).

Therefore, according to the above conceptual consideration, the ICD-11 model demonstrates some superiority over the DSM-5 model in terms of structural validity within the theoretical integration matrix provided by the CPM model. The DSM-5 model seems to fail with the 2nd and 3rd criteria formulated above. Namely, it contains a gap in covering the whole pathological personality space, with the underrepresentation of Delta-Plus characteristics. In turn, Psychoticism reveals problems with discriminant validity, overlapping with other DSM-5 domains (in particular Disinhibition and Antagonism) and makes the section between Alpha-Minus and Delta-Minus within the CPM excessively dense or overrepresented (see Figure 3). In contrast, the ICD-11 trait domains seem to cover cohesively the whole space of potentially problematic personality characteristics—from Delta-Minus to clockwise for Delta-Plus—fulfilling all three criteria formulated above (see Figure 3). Then, the ICD-11 model also embeds the Delta-Plus pole, and such assignment of Anankastia and Disinhibition as reflecting opposite poles of Delta suggests that they constitute a bipolar dimension. This is in line with both previous research on the ICD-11 model (7, 18–20), as well as the CPM assumption that Delta is an ambiguous dimension from a health-pathology perspective; as only Delta holds possibly pathological characteristics on both of its poles, with the other three metatraits (i.e., Alpha, Beta and Gamma) containing healthy and adaptive features on one (i.e., positive) pole as the opposite of the maladaptive characteristics located on the other (i.e., negative) one (see Figure 2).

Obviously, the above conceptual analyses need empirical verification, and the current research aims to provide a thorough test of these theoretical predictions. We examine the congruence between theoretical expectations and empirical locations within the CPM of the pathological personality traits from the ICD-11 and DSM-5 models, as well as from the joint six-domain model (32). As a point of departure, we verified the circumplex structure of the CPM itself, as well as the very possibility of locating each of the traits from the ICD-11, DSM-5, and six-domain models as external variables within the CPM.

Additionally, also the FFM dimensions were included in our study. The first purpose of that was to test the ICD-11 and DSM-5 Pathological Big Five models in reference to the FFM dimensions of normal personality. The second goal was to replicate previous findings concerning the CPM vs. FFM relationships, as well as to provide a comprehensive picture of relationships between all three Big Five models (FFM, DSM-5, and ICD-11) within the CPM space.

## Methods

### Participants and Procedure

Two studies were conducted. The sample in Study 1 consisted of 242 adults (52.9% female; *M*_age_ = 30.63 years, *SD*_age_ = 11.82 years), whereas participants in Study 2 were 355 adults (50.1% female; *M*_age_ = 29.97 years, *SD*_age_ = 12.26 years) mostly from central Poland. The research was conducted using a self-report paper-and-pencil method, with the assistance of trained psychology students. In both studies, similar sets of questionnaires were used, namely the Personality Inventory for ICD-11, the Personality Inventory for DSM-5 and the Circumplex of Personality Metatraits Questionnaire—Short Form^1^. Additionally, in Study 1 the Big Five Inventory-2 was administered in order to assess the FFM traits. Therefore, Study 2 is a replication of Study 1. For the sake of this methodological analogy, the methods and results of both studies were presented parallel rather than in sequence.

The research complied with the recommendations of the Commission of Ethics and Bioethics at the Cardinal Stefan Wyszyński University in Warsaw. The institutional guidelines for self-report questionnaire research on adults did not require formal approval by the Commission for this study. All participants provided their oral consent.

### Measures

#### Personality Inventory for ICD-11 (PiCD)

The PiCD [(17); Polish adaptation: (52)] is a 60-item self-report measure designed to assess the dimensional trait model proposed for the ICD-11 (2) containing five broad personality domains: Negative Affectivity, Disinhibition, Detachment, Dissociality, and Anankastia. Each domain contains 12 items rated on a 5-point Likert scale from 1 (*strongly disagree*) to 5 (*strongly agree*). In previous studies, the PiCD has been found to show adequate psychometric properties [e.g., convergent, discriminant, and structural validity; (17, 18)]. The Cronbach's alpha coefficients for the five PiCD scales in the current samples ranged from 0.72 to 0.88 (*M*_α_ = 0.82) in Study 1, and from 0.79 to 0.86 (*M*_α_ = 0.83) in Study 2 (see Supplementary Material).

#### Personality Inventory for DSM-5 (PID-5)

The PID-5 [(15); Polish adaptation: (53)] is a 220-item self-report measure capturing 25 traits across five domains of pathological personality according to Criterion B of the DSM-5 AMPD (1): Negative Affectivity, Detachment, Antagonism, Disinhibition, and Psychoticism. Items are rated on a 4-point Likert scale from 0 (*very false or often false*) to 3 (*very true or often true*). The PID-5 has been found to show adequate psychometric properties across clinical and non-clinical samples (15, 54, 55). The current study used only five domain-level scales (the 25 facets subscales were dropped) and these domain scores were calculated in line with the original five-factor structure of the PID-5 (15). Moreover, on the basis of the PID-5 items, we employed the algorithm with a 36-item modified PID5BF+ scoring key for the 6-domain model (6 items per domain) provided by Bach et al. (32). Reliability of original PID-5 domain scales, as measured by Cronbach's alpha coefficients, ranged from 0.87 to 0.95 (*M*_α_ = 0.93) in Study 1, and from 0.90 to 0.95 (*M*_α_ = 0.94) in Study 2. Reliabilities for the six-domain model ranged from 0.66 to 0.80 (*M*_α_ = 0.74) in Study 1, and from 0.65 to 0.77 (*M*_α_ = 0.74) in Study 2 (see Supplementary Material).

#### Circumplex of Personality Metatraits Questionnaire (CPM-Q-SF)

The CPM-Q-SF (10) is a 72-item inventory capturing a variety of human behaviors, feelings, and thoughts to assess eight metatraits according to the CPM: Alpha-Plus/Stability, Alpha-Minus/Disinhibition, Beta-Plus/Plasticity, Beta-Minus/Passiveness, Gamma-Plus/Integration, Gamma-Minus/Disharmony, Delta-Plus/Self-Restraint, and Delta-Minus/Sensation Seeking (for the meaning of the metatraits see Table 1). Each metatrait contains nine items rated on a 5-point Likert scale from 1 (*completely disagree*) to 5 (*completely agree*). In previous studies, the CPM-Q-SF has been found to show adequate psychometric properties (10, 49, 56). The Cronbach's alpha coefficients for the eight CPM-Q-SF scales in the current samples ranged from 0.69 to 0.86 (*M*_α_ = 0.78) in Study 1, and from 0.70 to 0.87 (*M*_α_ = 0.79) in Study 2 (see Supplementary Material).

#### The Big Five Inventory-2 (BFI-2)

The BFI-2 (57) is a revised version of the Big Five Inventory, one of the most commonly used measures of the FFM (58). The BFI-2 contains 60 items assessing Neuroticism, Extraversion, Agreeableness, Conscientiousness, and Openness as well as the total of their 15 facets. Each domain contains 12 items and each facet is measured by four items rated on a 5-point Likert scale from 1 (*strongly disagree*) to 5 (*strongly agree*). In previous studies, the BFI-2 has been found to show adequate psychometric properties, allowing assessment of key personality traits in a concise and comprehensible way (57). Reliability of these scales in the current sample (Study 1) ranged from 0.82 to 0.90 (*M*_α_ = 0.87) for the domain scales, and from 0.64 to 0.86 (*M*_α_ = 0.76) for the facet subscales (see Supplementary Material).

### Statistical Approach

To provide a full test of the hypotheses regarding relationships of the ICD-11, DSM-5, and FFM traits with the CPM metatraits, we followed the three-step procedure for analysis of circumplex models (49). Importantly, each step is at the same time a prerequisite for the subsequent analyses. These three steps are: (1) verification of the CPM circumplex structure using a Structural Equation Model (SEM) based on the circular stochastic process model; (2) testing the possibility to locate external variables within the empirical circumplex of the CPM on the basis of the Structural Summary Method (SSM); and, finally, (3) testing congruence between theoretical expectations and empirical locations of whole ICD-11, DSM-5, and FFM models within the CPM structure using the Procrustes-based comparative procedure. The details of each step are fully described in Rogoza et al. (49) paper. The SEM and SSM were carried out in R in CircE (59) and circumplex (60) packages, while the Procrustes procedure was conducted in Orthosim 3 (61). To evaluate SEM, we used the commonly used criteria of good model fit: Comparative Fit Index (CFI), Tucker-Lewis Index (TLI) and Goodness of Fit Index (GFI) > 0.90, as well as Adjusted Goodness of Fit Index (AGFI) > 0.85, and Standardized Root Mean Squared Residual (SRMR) < 0.10 (62, 63), but with one exception. As the widely used Root Mean Square Error of Approximation (RMSEA) metric seems to produce artificially high estimates in circumplex models, we used a cutoff of < 0.13 as suggested by Gurtman and Pincus (64). For evaluation of SSM results, we used the commonly used criteria: model fit estimate > 0.80; elevation < 0.15 (i.e., indicating no presence of the general factor) and amplitude > 0.15 (i.e., distinctiveness of profile) that denote that an external variable can be meaningfully located within a given circumplex model (60). Finally, at the third step, analyses with the application of Procrustes rotation yield two sorts of congruence coefficients. First, overall solution congruence (i.e., does the whole model fit within the hypothesized location), and, second, specific congruence (i.e., does a specific variable fit within the hypothesized location). Congruence coefficients above 0.85 indicate an acceptable fit between the empirical and theoretical matrices, and those above 0.95 indicate excellent congruence (61, 65–67). Additionally, explained variance coefficients (*R*^2^) were calculated for indicators of each trait and metatraits to assess their communality in the joint two-factor CPM space.

Of note, at the third step, the procedure makes it possible to compare an empirically obtained factor matrix with a theoretically predicted structure (target). In the current research, the target matrix represents the theoretically predicted locations of the CPM, ICD-11, DSM-5, and FFM variables within the CPM circumplex space (see Figures 1, 2). The theoretical locations were acquired by assigning an angle to each variable as the commonly used specification of locations within circumplex models (65), and then estimating factor loadings on two factors based on a given angle. These factors represent the two major axes of the CPM space (i.e., the bipolar metatraits Alpha and Beta), with loadings being the sines and cosines of particular angles. The specification begins from Beta-Plus, which has an angle of 0° and loadings resulting from this angle: 0.00 on the first factor (Alpha) and 1.00 on the second one (Beta). Then, it is continued counterclockwise for the other seven metatraits, as well as the ICD-11, DSM-5, and FFM traits. The empirical factor matrices were obtained using Principal Axis Factoring (PAF) of a given set of jointly analyzed variables with target Procrustes rotation; in this way, two factors as the basic axes of the CPM model were extracted. The procedure of testing congruence between empirical locations and theoretical expectations within circumplex structure was run three times in each sample taking the three different sets of variables. In Study 1 it was: CPM, FFM, and ICD-11 variables (Analysis A); CPM, FFM, and DSM-5 variables (Analysis B); CPM, FFM, and joint six-domains variables (Analysis C). The same was applied for Study 2 except for the FFM variables that were not present in this study.

## Results

Full set of descriptive statistics (i.e., mean and standard deviation), reliability estimates for scale scores, and (inter)correlations among variables used in both Study 1 and Study 2 can be found in Supplemental Material.

### The ICD-11, DSM-5, and Six-Domain Models of Pathological Traits in Context of the FFM Normal Personality Structure

At the outset, the correlation analysis was performed testing the ICD-11 and DSM-5 pathological domains in reference to the Big Five/FFM dimensions of normal personality. Table 2 depicts obtained correlation coefficients additionally including six-domain algorithm scores as well as facets of the FFM domains.

**Table 2:** Correlations of FFM domains and facets with ICD-11 and DSM-5 domains in Study 1 (*N* = 242).

		**ICD-11 model**					**DSM-5 model**					**Common six-domain model**					
		**NA**	**DT**	**DL**	**DN**	**AK**	**NA**	**DT**	**AN**	**DN**	**PS**	**NA**	**DT**	**AN**	**DN**	**AK**	**PS**
Domains	NEUROTICISM	0.74	0.26	−0.02	0.32	0.07	0.66	0.50	0.15	0.08	0.27	0.65	0.31	0.05	0.34	0.22	0.18
	EXTRAVERSION	−0.37	−0.63	0.27	−0.08	−0.14	−0.32	−0.54	0.21	0.10	−0.11	−0.25	−0.45	0.14	−0.12	−0.13	−0.02
	AGREEABLENESS	−0.18	−0.23	−0.56	−0.35	0.13	−0.26	−0.30	−0.53	−0.24	−0.35	−0.13	−0.29	−0.42	−0.34	−0.18	−0.27
	CONSCIENTIOUSNESS	−0.26	−0.14	−0.19	−0.66	0.29	−0.27	−0.24	−0.29	−0.53	−0.36	−0.16	−0.16	−0.23	−0.55	0.15	−0.31
	OPENNESS	−0.14	−0.30	0.08	−0.03	0.02	−0.17	−0.28	0.09	0.05	0.01	−0.14	−0.30	0.04	−0.04	−0.11	0.07
Facets	N_Anxiety	0.67	0.30	−0.10	0.16	0.19	0.55	0.46	0.06	−0.04	0.20	0.57	0.31	0.01	0.20	0.20	0.12
	N_Depression	0.66	0.38	−0.08	0.15	0.19	0.61	0.58	0.01	−0.04	0.22	0.58	0.41	−0.04	0.21	0.24	0.11
	N_Emotional Volatility	0.54	−0.04	0.12	0.51	−0.21	0.52	0.22	0.31	0.30	0.28	0.49	0.08	0.17	0.46	0.11	0.22
	E_Sociability	−0.29	−0.63	0.23	−0.01	−0.16	−0.22	−0.47	0.22	0.09	−0.06	−0.17	−0.43	0.14	−0.08	−0.11	0.01
	E_Assertiveness	−0.35	−0.45	0.36	−0.11	−0.06	−0.28	−0.38	0.28	0.06	−0.06	−0.22	−0.30	0.21	−0.15	−0.07	0.00
	E_Energy Level	−0.33	−0.59	0.09	−0.09	−0.14	−0.35	−0.58	0.04	0.11	−0.18	−0.30	−0.48	0.01	−0.07	−0.17	−0.07
	A_Compassion	0.04	−0.21	−0.52	−0.28	0.16	−0.05	−0.21	−0.44	−0.27	−0.31	0.06	−0.25	−0.36	−0.26	−0.07	−0.27
	A_Respectfulness	−0.14	−0.03	−0.42	−0.35	0.23	−0.21	−0.15	−0.43	−0.24	−0.28	−0.11	−0.12	−0.33	−0.30	−0.14	−0.20
	A_Trust	−0.32	−0.30	−0.42	−0.23	−0.06	−0.38	−0.38	−0.43	−0.09	−0.27	−0.27	−0.33	−0.34	−0.29	−0.22	−0.18
	C_Organization	−0.15	−0.11	−0.17	−0.57	0.29	−0.16	−0.14	−0.26	−0.51	−0.27	−0.07	−0.06	−0.17	−0.50	0.20	−0.24
	C_Productiveness	−0.35	−0.18	−0.08	−0.53	0.13	−0.39	−0.35	−0.23	−0.36	−0.31	−0.30	−0.26	−0.18	−0.48	0.02	−0.23
	C_Responsibility	−0.18	−0.08	−0.24	−0.61	0.33	−0.16	−0.14	−0.28	−0.48	−0.36	−0.05	−0.10	−0.27	−0.43	0.14	−0.35
	O_Intellectual Curiosity	−0.19	−0.21	0.13	0.04	−0.07	−0.18	−0.20	0.13	0.18	0.01	−0.16	−0.20	0.07	0.01	−0.18	0.05
	O_Aesthetic Sensitivity	0.02	−0.16	−0.09	−0.02	0.07	−0.03	−0.12	−0.05	−0.04	0.06	−0.02	−0.19	−0.08	0.01	−0.06	0.10
	O_Creative Imagination	−0.22	−0.39	0.21	−0.08	0.01	−0.23	−0.38	0.18	0.01	−0.07	−0.20	−0.34	0.14	−0.12	−0.03	0.00

*NA, Negative Affectivity; DT, Detachment; DN, Disinhibition; DL, Dissociality; AN, Antagonism; AK, Anankastia; PS, Psychoticism; N, Neuroticism; E, Extraversion; A, Agreeableness; C, Conscientiousness; O, Openness to Experiences. Correlations > |0.12| are significant at p < 0.05 (two-tailed)*.

The ICD-11 traits showed a clear and meaningful pattern of associations when related to the FFM traits, reflecting good convergent and discriminant validity. Namely, Negative Affectivity, Detachment, Dissociality, as well as Disinhibition vs. Anankastia domains showed medium to strong empirical convergence with Neuroticism, Extraversion, Agreeableness, and Conscientiousness, respectively. However, the correlations of Anankastia with Conscientiousness were weaker (*r* = 0.29), due to the low correlations between Anankastia and the most proactive facet of Conscientiousness (i.e., Productiveness), although still the strongest one compared to the other FFM domains. Moreover, attention also attracts the correlation of Disinhibition with the emotional lability facet of Neuroticism.

The DSM-5 domains also revealed a pattern of relationships with FFM traits that was relatively convergent with expectations, however, it was not as valid and clear as in the case of the ICD-11 traits. Negative Affectivity, Antagonism, and Disinhibition showed good convergent and discriminant validity, but Detachment and Psychoticism did not. As far as the strong cross-correlation with Neuroticism (and especially with its Depression facet) revealed by Detachment is understandable, given its common facets with Negative Affectivity (15), Psychoticism showed zero correlations with the Openness domain (which is supposed to be its counterpart), as well as with all of its three facets. Instead, Psychoticism revealed negative correlations with Conscientiousness, Agreeableness, as well as positive (although weaker) correlations with Neuroticism domains and facets. Also, the six-domain algorithm revealed some problems with convergent and discriminant validity, particularly in the case of Psychoticism and Anankastia. Regarding Psychoticism, its problems are very analogous to the above-mentioned. In turn, Anankastia showed a minor correlation with Conscientiousness, being more strongly related with Neuroticism (particularly with Depression facet) and even (negatively) with Agreeableness. Summing up, the obtained results indicated the ICD-11 model as showing a generally clearer and more theoretically cohesive pattern of relationships with the FFM model than DSM-5 and six-domain models.

### ICD-11, DSM-5, and Six-Domain Models of Pathological Traits (and FFM Normal Traits) in Context of the CPM Model of Personality Structure

#### Step 1: Testing the CPM Structure

First, we tested whether the CPM meets the criteria of the circumplex model with equal spacing and equal communalities. The results obtained in Study 1 supported the circumplex structure of the model: χ^2^(24) = 70.28; *p* < 0.001; CFI = 0.948; TLI = 0.940; GFI = 0.955; AGFI = 0.932; RMSEA = 0.089; SRMR = 0.080. These results were replicated in Study 2, with the following fit indices: χ^2^(24) = 104.61; *p* < 0.001; CFI = 0.941; TLI = 0.931; GFI = 0.947; AGFI = 0.920; RMSEA = 0.097; SRMR = 0.086. Therefore, circumplex internal structure of the CPM model was confirmed in both studies, based on the suggestion by Gurtman and Pincus [(64); see: (49)], that for circumplex models the cutoff of RMSEA < 0.13 can be acceptable. Other model fit indicators met the usual criteria described above. Such results provide the possibility to move onto the next step – to locate external variables within the circumplex space.

#### Step 2: Testing Possibilities of Location of the ICD-11, DSM-5, Six-Domain, and FFM Traits Within the CPM Model

The results of the SSM obtained in Study 1 (see Table 3) suggest that all of the traits of the ICD-11, DSM-5, six-domain model, and FFM were well-fitted to the circumplex of the CPM model. All of the amplitude values were above 0.15, suggesting that each profile is clearly distinct, and all values of elevation were below 0.15, suggesting no or very little influence of the general factor (as CPM does not assume the presence of any sort of general factor). Also, given that model fit values for all traits were above 0.88, all of the external variables' correlation profiles were well-fitted to the cosine curve reflecting the CPM model.

**Table 3:** Structural summary profiles of the ICD-11, DSM-5, six-domain model, and FFM traits projected on the circumplex space of the CPM for Study 1 (left side) and Study 2 (right side).

**Trait**	**Elevation**		**Amplitude**		**Fit**	
ICD-11						
Negative Affectivity	0.05 [0.01, 0.09]	0.04 [0.01, 0.08]	0.45 [0.35, 0.55]	0.51 [0.45, 0.57]	0.959	0.969
Detachment	0.01 [−0.03, 0.04]	0.02 [−0.01, 0.05]	0.49 [0.40, 0.58]	0.46 [0.39, 0.54]	0.991	0.990
Dissociality	0.10 [0.06, 0.14]	0.07 [0.03, 0.10]	0.35 [0.27, 0.43]	0.39 [0.31, 0.46]	0.950	0.945
Disinhibition	0.03 [−0.00, 0.08]	0.00 [−0.03, 0.03]	0.46 [0.38, 0.54]	0.51 [0.44, 0.59]	0.951	0.981
Anankastia	0.05 [0.00, 0.10]	0.07 [0.04, 0.10]	0.31 [0.23, 0.40]	0.35 [0.27, 0.43]	0.883	0.935
DSM-5						
Negative Affectivity	0.10 [0.07, 0.14]	0.06 [0.03, 0.10]	0.47 [0.38, 0.56]	0.48 [0.41, 0.55]	0.976	0.977
Detachment	0.06 [0.03, 0.09]	0.04 [0.01, 0.07]	0.54 [0.46, 0.61]	0.57 [0.50, 0.63]	0.969	0.988
Antagonism	0.12 [0.08, 0.16]	0.07 [0.04, 0.10]	0.39 [0.32, 0.47]	0.44 [0.37, 0.50]	0.948	0.980
Disinhibition	0.04 [0.01, 0.08]	0.03 [−0.00, 0.05]	0.45 [0.37, 0.53]	0.49 [0.42, 0.56]	0.955	0.965
Psychoticism	0.11 [0.07, 0.14]	0.06 [0.03, 0.09]	0.32 [0.23, 0.40]	0.33 [0.26, 0.41]	0.986	0.942
Common six-domain model						
Negative Affectivity	0.07 [0.03, 0.10]	0.05 [0.02, 0.09]	0.39 [0.30, 0.49]	0.30 [0.22, 0.38]	0.968	0.933
Detachment	0.03 [0.00, 0.07]	0.05 [0.01, 0.08]	0.48 [0.39, 0.55]	0.43 [0.35, 0.50]	0.991	0.996
Antagonism	0.09 [0.05, 0.13]	0.06 [0.02, 0.09]	0.31 [0.22, 0.40]	0.34 [0.26, 0.42]	0.953	0.982
Disinhibition	0.06 [0.03, 0.10]	0.05 [0.03, 0.08]	0.38 [0.29, 0.47]	0.44 [0.37, 0.51]	0.952	0.980
Anankastia	0.11 [0.07, 0.14]	0.08 [0.05, 0.12]	0.25 [0.15, 0.34]	0.06 [0.01, 0.17]	0.928	0.655
Psychoticism	0.09 [0.06, 0.13]	0.08 [0.04, 0.11]	0.24 [0.16, 0.33]	0.26 [0.19, 0.34]	0.990	0.917
FFM						
Neuroticism	0.01 [−0.03, 0.04]	-	0.54 [0.46, 0.62]	-	0.959	-
Extraversion	0.05 [0.02, 0.08]	-	0.66 [0.59, 0.73]	-	0.988	-
Agreeableness	0.01 [−0.02, 0.04]	-	0.50 [0.43, 0.58]	-	0.972	-
Conscientiousness	0.03 [−0.01, 0.06]	-	0.45 [0.37, 0.53]	-	0.951	-
Openness	0.02 [−0.01, 0.06]	-	0.52 [0.44, 0.60]	-	0.959	-

*The lower and upper 95% confidence intervals for elevation and amplitude estimates are given in parenthesis*.

These results were replicated (without FFM traits) in Study 2—besides one exception (i.e., Anankastia from the 6-domain model), all of the external variables' correlation profiles were well-fitted to the cosine curve reflecting the CPM model (see Table 3). All model fit values > 0.90; elevation > 0.15 (suggesting no general factor) and amplitude > 0.15 (reflecting distinctiveness of profiles) indicate that each of the external variables can be meaningfully located within the CPM. Therefore, results obtained in both studies almost fully confirmed the possibility for the analyses in step 3, that is, to precisely test the hypotheses concerning the location of all traits from the given model within the joint circumplex space. With regard to one variable that did not meet the criteria of step 2 in Study 2—i.e., Anankastia from the 6-domain model revealing low fit and distinctiveness—we decided to continue the analyses in step 3 to provide more precise insight into the level of its incongruence, although in this case we could withhold analyses on the second step.

#### Step 3. Testing the Congruence Between Empirical and Theoretical Locations of the ICD-11, DSM-5, Six-Domain, and FFM Models Within the CPM Space

At this stage, we applied Procrustes-based comparative analyses (described in the Statistical Approach subsection) to precisely test the hypotheses concerning the location of the ICD-11, DSM-5, and common six-domain models of pathological traits (as well as the FFM model of normal traits in Study 1) within the CPM. In Study 1 PAF led to the extraction of two factors with eigenvalues >1 and accounting for 56.8% (Analysis A: CPM, ICD-11, and FFM), 59.0% (Analysis B: CPM, DSM-5, and FFM), and 53.3% (Analysis C: CPM, six-domain model, and FFM) of the variance. The eigenvalues of the first two factors were 5.75 and 4.48 (Analysis A), 6.17 and 4.45 (Analysis B), and 5.93 and 4.19 (Analysis C). Correlation coefficients within the obtained pairs of regression-based factor scores (after Varimax rotation) were−0.01, 0.00, and 0.02 (for the first, second, and third analysis, respectively), which justifies treating them as orthogonal axes of the circumplex space.

**Figure 4:**
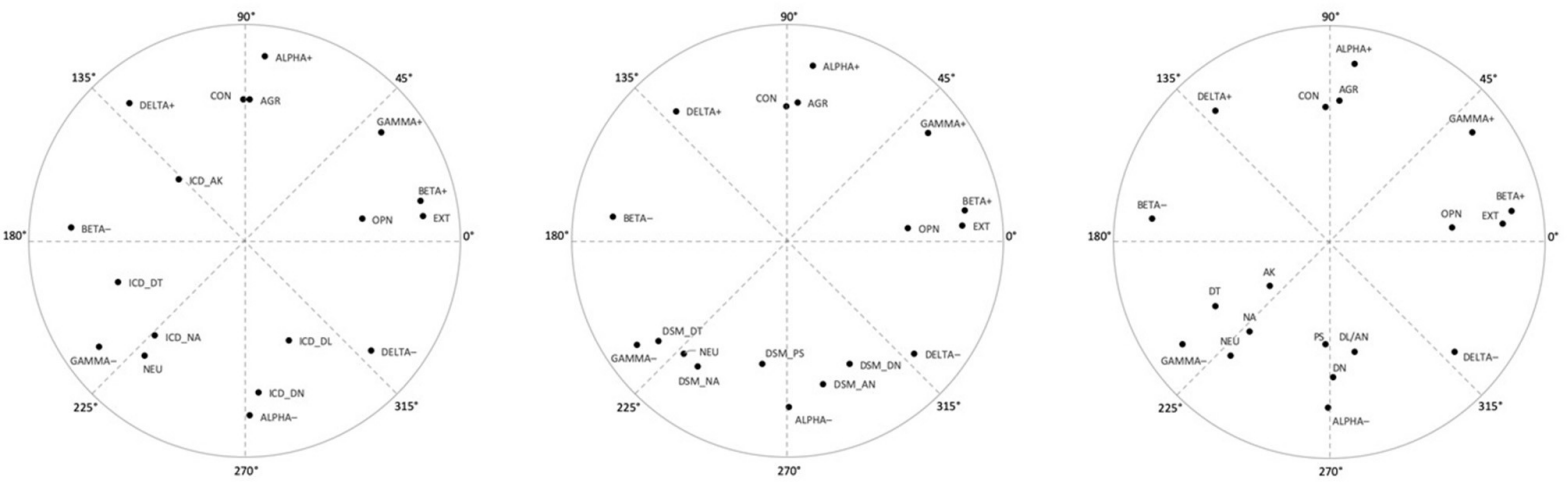
Factor loading plots in Study 1 (*N* = 242) for: (Analysis A) CPM metatraits, FFM and ICD-11 traits; (Analysis B) CPM metatraits, FFM and DSM-5 traits; and (Analysis C) CPM metatraits, FFM and the six-domain model traits (placed at left, middle, and right, respectively). NA, Negative Affectivity; DT, Detachment; DN, Disinhibition; DL, Dissociality; AN, Antagonism; AK, Anankastia; PS, Psychoticism; NEU, Neuroticism; EXT, Extraversion; AGR, Agreeableness; CON, Conscientiousness; OPN, Openness to Experiences; + denotes the positive pole of a metatrait; – denotes the negative pole of a metatrait.

Table 4 depicts the theoretical (target) and empirical matrices observed in three analyses, together with congruence coefficients between these matrices for overall models as well as for each specific variable (see also Figure 4 for graphical presentation of the results). In terms of the overall model, including both major factors (axes), the congruence coefficients obtained by comparing the observed and target matrices exceeded 0.95 in Analysis A and Analysis B, whereas in Analysis C (six-domain and FFM models) these values were below 0.95, although above 0.90. Regarding CPM and FFM variables, all congruencies were 0.98 or higher and in many cases reached 1.00 (in all three analyses), which indicate an excellent fit to the theoretical assumptions. Also satisfactory in most cases, although visibly lower, were values obtained for ICD-11, DSM-5, and the six-domain model. Regarding the ICD-11 model (Analysis A), worth noting is the perfect match of the Anankastia domain. On the other hand, one domain revealed low congruence, namely Disinhibition with a coefficient below 0.85 and a deviation of 39.7°. The DSM-5 model fared somewhat worse (Analysis B), as two of its traits obtained congruence coefficients below 0.85. They were Psychoticism and Detachment with deviations of 38.3 and 39.7°, respectively. The worst congruence with the expected location revealed traits from the six-domain model in Analysis C, with a trivial fit of Anankastia (displacement of 81.3°), a low fit of Disinhibition (displacement of 45.0°), and an only slightly above 0.85 fit of Detachment (displacement of 28.7°). Nevertheless, the other three traits from this model revealed satisfactory congruencies.

**Table 4:** Target and obtained factor matrices with corresponding CPM angles, explained variances, and congruence coefficients for comparing target and obtained factor loadings of CPM metatraits, ICD-11 and DSM-5 psychopathological and FFM normal trait-domains in three analyses conducted in Study 1 (*N* = 242).

		**Target matrix**			**Analysis A**					**Analysis B**					**Analysis C**				
					**ICD-11 model**					**DSM-5 model**					**Common six-domains model**				
		**Θ**	**F1**	**F2**	**F1**	**F2**	***R^**2**^***	**Congr**.	**Θ**	**F1**	**F2**	***R^**2**^***	**Congr**.	**Θ**	**F1**	**F2**	***R^**2**^***	**Congr**.	**Θ**
CPM	Delta-Plus	135	0.71	−0.71	0.63	−0.53	0.67	1.00	129.4	0.60	−0.51	0.62	1.00	130.6	0.60	−0.52	0.64	1.00	129.0
	Alpha-Plus	90	1.00	0.00	0.85	0.10	0.73	0.99	82.5	0.81	0.12	0.66	0.99	81.9	0.82	0.12	0.70	0.99	79.7
	Gamma-Plus	45	0.71	0.71	0.50	0.64	0.66	0.99	37.3	0.50	0.65	0.68	0.99	37.8	0.50	0.66	0.68	0.99	35.5
	Beta-Plus	0	0.00	1.00	0.18	0.82	0.71	0.98	11.3	0.14	0.82	0.69	0.99	9.9	0.14	0.84	0.72	0.99	7.8
	Delta-Minus	315	−0.71	0.71	−0.51	0.59	0.60	1.00	318.4	−0.52	0.59	0.62	1.00	319.2	−0.51	0.58	0.59	1.00	317.0
	Alpha-Minus	270	−1.00	0.00	−0.81	0.03	0.65	1.00	271.1	−0.77	0.01	0.59	1.00	270.7	−0.77	0.00	0.59	1.00	267.9
	Gamma-Minus	225	−0.71	−0.71	−0.49	−0.67	0.69	0.99	215.2	−0.48	−0.69	0.71	0.98	215.2	−0.48	−0.67	0.69	0.99	214.0
	Beta-Minus	180	0.00	−1.00	0.06	−0.80	0.65	1.00	174.8	0.11	−0.80	0.65	0.99	172.7	0.10	−0.81	0.67	0.99	171.3
ICD-11/DSM-5	Negative Affectivity	225	−0.71	−0.71	−0.44	−0.41	0.36	1.00	225.8	−0.58	−0.41	0.51	0.98	235.3	−0.42	−0.36	0.31	1.00	228.0
	Detachment	180	0.00	−1.00	−0.19	−0.58	0.38	0.95	197.3	−0.46	−0.59	0.56	0.79	218.3	−0.30	−0.52	0.36	0.86	208.7
	Dissociality/Antagonism	270	−1.00	0.00	−0.46	0.21	0.25	0.91	293.4	−0.66	0.17	0.46	0.97	284.5	−0.51	0.12	0.27	0.97	281.4
	Disinhibition	315	−0.71	0.71	−0.70	0.07	0.50	0.78	275.3	−0.57	0.29	0.41	0.95	296.9	−0.63	0.02	0.39	0.73	270.0
	Anankastia	135	0.71	−0.71	0.28	−0.30	0.17	1.00	136.1						−0.21	−0.27	0.12	0.12	216.3
	Psychoticism	292.5	−0.92	0.38						−0.57	−0.11	0.34	0.83	259.4	−0.48	−0.01	0.23	0.92	267.2
FFM	Neuroticism	225	−0.71	−0.71	−0.53	−0.46	0.49	1.00	228.2	−0.52	−0.47	0.48	1.00	228.2	−0.53	−0.45	0.48	1.00	227.7
	Extraversion	0	0.00	1.00	0.11	0.83	0.71	0.99	6.5	0.07	0.81	0.66	1.00	5.5	0.08	0.80	0.65	1.00	4.0
	Agreeableness	90	1.00	0.00	0.65	0.03	0.43	1.00	86.5	0.64	0.05	0.42	1.00	86.0	0.65	0.05	0.43	1.00	83.8
	Conscientiousness	90	1.00	0.00	0.65	0.00	0.43	1.00	89.7	0.62	0.00	0.38	1.00	90.1	0.62	−0.01	0.39	1.00	89.1
	Openness	0	0.00	1.00	0.10	0.55	0.31	0.98	9.5	0.06	0.56	0.31	0.99	6.1	0.06	0.57	0.33	0.99	4.4
	Factor/Overall congruence				0.97	0.95		0.98		0.97	0.96		0.97		0.92	0.94		0.92	

*The target matrix is based on the hypothesized circumplex structure shown in Figure 2. Θ, angles in degrees; F1 and F2, factors; Congr., Congruence coefficients*.

The above results were generally replicated in Study 2. PAF led to the extraction of two factors with eigenvalues >1 and accounting for 61.4% (Analysis A: CPM and ICD-11), 64.4% (Analysis B: CPM and DSM-5), and 54.7% (Analysis C: CPM and six-domain model) of the variance. The eigenvalues of the first two factors were 4.61 and 3.37 (Analysis A), 5.14 and 3.24 (Analysis B), and 4.57 and 3.08 (Analysis C). Correlation coefficients within the obtained pairs of regression-based factor scores (after varimax rotation) were −0.18, −0.18, and 0.35 (for the first, second, and third analysis, respectively), which seems to support treating them as orthogonal axes of the circumplex only in the first two cases.

**Figure 5:**
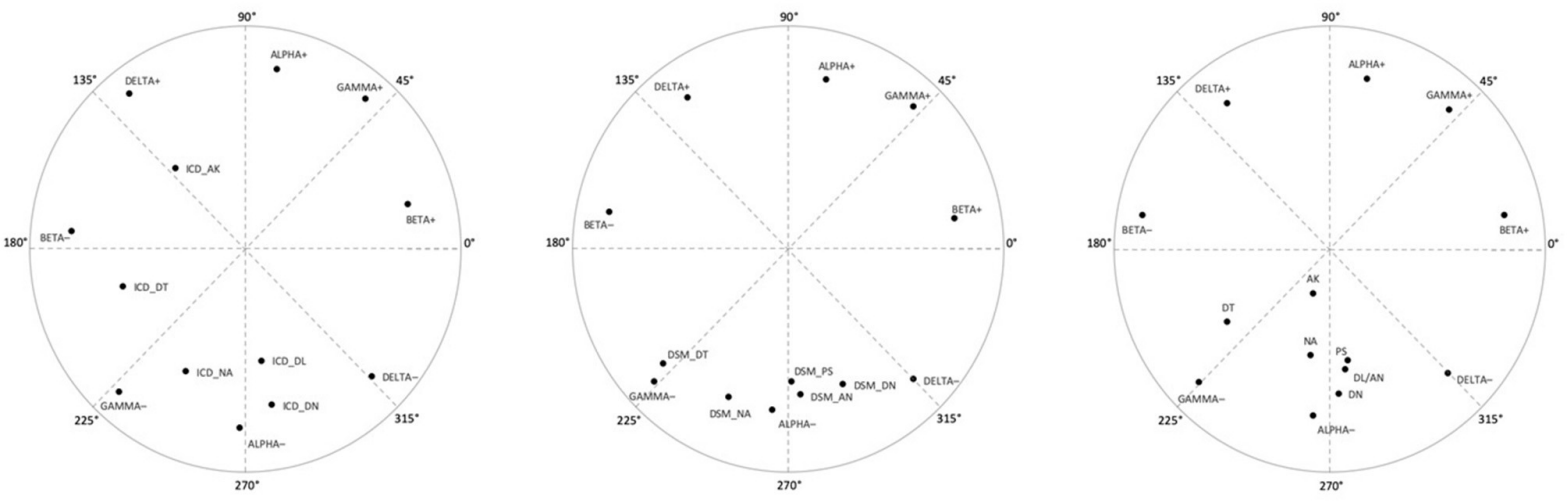
Factor loading plots in Study 2 (*N* = 355) for: (Analysis A) CPM metatraits and ICD-11 traits; (Analysis B) CPM metatraits and DSM-5 traits; and (Analysis C) CPM metatraits and the six-domain model traits (placed at left, middle, and right, respectively). NA, Negative Affectivity; DT, Detachment; DN, Disinhibition; DL, Dissociality; AN, Antagonism; AK, Anankastia; PS, Psychoticism; + denotes the positive pole of a metatrait; – denotes the negative pole of a metatrait.

For the results of comparison of target and empirical matrices obtained in the three analyses of Study 2, see Table 5 and Figure 5 (in a juxtaposition with Figure 3). In terms of the overall model, the congruence coefficients exceeded 0.95 in Analysis A and Analysis B, whereas in Analysis C (CPM and six-domain model) they range only between 0.85 (overall coefficient) and 0.90 (for factor 1). The CPM metatraits showed a very good fit in all cases (congruence coefficients >0.95), with no serious deviation from the expected locations. Regarding traits from the ICD-11 model (Analysis A), almost all congruence coefficients were above 0.95 indicating a very good fit of the observed structure to the theoretically predicted, with a perfect fit for Anankastia. The only exception was the Disinhibition domain, which again revealed a coefficient below 0.83 and a shift of 34.7° toward Alpha-Minus. Slightly more serious deviations were found in the case of the DSM-5 model traits (Analysis B). Namely, Detachment revealed a congruence of 0.74 and displacement of 42.7° (again toward Gamma-Minus), and only one of the other traits had a coefficient above 0.95 (i.e., Antagonism which fit perfectly). However, worth noting is that this time (i.e., in contrast to Study 1) Psychoticism showed acceptable fit with a relatively low range of displacement (i.e., 20.1°). In contrast, almost all variables from the six-domain algorithm (Analysis C) revealed congruence coefficients below 0.85 indicating a poor fit to the theoretical predictions (displacements ranged between 31.0° of Detachment to 110.5° of Anankastia). Two exceptions with very good fit were Psychoticism and Antagonism, whereas the worst fit occurred for Anankastia with a congruence −0.42, and the observed location between Gamma-Minus and Alpha-Minus rather than in Delta-Plus.

**Table 5:** Target and obtained factor matrices with corresponding CPM angles, explained variances, and congruence coefficients for comparing target and obtained factor loadings of CPM metatraits, ICD-11 and DSM-5 psychopathological trait-domains in three analyses conducted in Study 2 (*N* = 355).

		**Target matrix**			**Analysis A**					**Analysis B**					**Analysis C**				
					**ICD-11 model**					**DSM-5 model**					**Common six-domains model**				
		**Θ**	**F1**	**F2**	**F1**	**F2**	***R^**2**^***	**Congr**.	**Θ**	**F1**	**F2**	***R^**2**^***	**Congr**.	**Θ**	**F1**	**F2**	***R^**2**^***	**Congr**.	**Θ**
CPM	Delta-Plus	135	0.71	−0.71	0.69	−0.53	0.76	0.99	126.8	0.67	−0.46	0.66	0.98	125.0	0.65	−0.46	0.64	0.98	120.5
	Alpha-Plus	90	1.00	0.00	0.80	0.15	0.67	0.98	79.2	0.75	0.18	0.59	0.97	77.2	0.76	0.18	0.61	0.97	71.9
	Gamma-Plus	45	0.71	0.71	0.67	0.56	0.76	1.00	49.5	0.63	0.58	0.73	1.00	47.7	0.62	0.56	0.70	1.00	43.9
	Beta-Plus	0	0.00	1.00	0.20	0.76	0.62	0.97	14.3	0.13	0.77	0.61	0.99	9.7	0.15	0.81	0.67	0.98	5.8
	Delta-Minus	315	−0.71	0.71	−0.57	0.59	0.67	1.00	315.8	−0.59	0.58	0.68	1.00	314.8	−0.56	0.55	0.62	1.00	310.0
	Alpha-Minus	270	−1.00	0.00	−0.80	−0.02	0.63	1.00	267.7	−0.73	−0.07	0.54	1.00	265.2	−0.75	−0.07	0.56	1.00	260.5
	Gamma-Minus	225	−0.71	−0.71	−0.64	−0.58	0.74	1.00	227.3	−0.60	−0.61	0.73	1.00	224.7	−0.60	−0.59	0.71	1.00	221.2
	Beta-Minus	180	0.00	−1.00	0.08	−0.80	0.65	0.99	173.6	0.16	−0.82	0.70	0.98	169.2	0.15	−0.85	0.75	0.98	165.4
ICD-11/DSM-5	Negative Affectivity	225	−0.71	−0.71	−0.55	−0.27	0.37	0.95	243.4	−0.67	−0.27	0.53	0.92	248.3	−0.48	−0.08	0.24	0.81	256.6
	Detachment	180	0.00	−1.00	−0.17	−0.56	0.34	0.96	196.1	−0.52	−0.57	0.60	0.74	222.7	−0.33	−0.46	0.32	0.82	211.0
	Dissociality/Antagonism	270	−1.00	0.00	−0.50	0.08	0.25	0.99	278.3	−0.66	0.06	0.44	1.00	275.8	−0.54	0.08	0.30	0.99	274.4
	Disinhibition	315	−0.71	0.71	−0.70	0.13	0.50	0.83	280.3	−0.61	0.26	0.44	0.93	293.2	−0.65	0.05	0.43	0.76	269.9
	Anankastia	135	0.71	−0.71	0.36	−0.32	0.23	1.00	131.3						−0.20	−0.07	0.04	−0.42	245.5
	Psychoticism	292.5	−0.92	0.38						−0.60	0.02	0.37	0.94	272.4	−0.50	0.09	0.25	0.98	275.6
	Factor/Overall congruence				0.97	0.95		0.97		0.96	0.96		0.96		0.90	0.88		0.85	

*The target matrix is based on the hypothesized circumplex structure shown in Figure 2. Θ, angles in degrees; F1 and F2, factors; Congr., Congruence coefficients*.

Summing up, the results obtained in both studies indicate a generally satisfactory fit of the observed variable structures to the theoretically assumed one. Therefore, the expectations regarding the location of the ICD-11 and DSM-5 models within the CPM can be deemed as overall confirmed. These congruencies were definitely the best in the case of the CPM and FFM models, and visibly lower, although generally also satisfactory in the case of the ICD-11 and DSM-5 models. The ICD-11 model revealed relatively better congruence coefficients than the DSM-5 model, with a perfect fit of Anankastia in both studies and an ambiguous fit of Psychoticism (i.e., low fit in Study 1 and acceptable in Study 2) to the expected location. The six-domains model revealed the poorest and generally low coefficients, especially in Study 2. Of note are also generally lower communalities of pathological traits with CPM structure, particularly in the case of the six-domain model, but also in the case of the ICD-11. Especially low *R*^2^ values revealed Anankastia from the six-domain model, although also Anankastia from the ICD-11 (PiCD) showed low communalities as well as lower correlations with FFM traits. However, these results are fully understandable as CPM metatraits are not directly or specifically personality disorder dimensions. The CPM is a general model of personality structure with a space for PD but also other forms of psychopathology or dysfunction (9, 48, 49, 56). Therefore, ICD-11 or DSM-5 traits are not reducible to the CPM metatraits, even though they were quite precisely located in the space defined by them, as evidenced by high congruence coefficients.

## Discussion

Both APA (1) and WHO (2) introduced dimensional models of PD in their newest editions of disorder classification systems (DSM-5 and ICD-11, respectively). These models are largely congruent with the crucial position of five broad domains (so-called Pathological Big Five), nevertheless, they also substantially differ in some elements. One of the most evident differences is related to the old disagreement between these two authoritative institutions, i.e., placement of schizotypic characteristics. As a result, the fifth dimension in DSM-5 is Psychoticism, while in ICD-11 it is Anankastia. Given the evidence supporting the value of both Psychoticism and Anankastia features to clinical psychological examination, and personality pathology itself, the current paper seeks to answer the question of which one is structurally better justified as the fifth trait in the Pathological Big Five. We proceeded to address this question by adopting the CPM model as a reference framework.

### Circumplex of Personality Metatraits Indicates Anankastia Rather Than Psychoticism

Specifically, we made an attempt to compare the DSM-5 and ICD-11 models with the lens of the CPM, which is a comprehensive model of personality structure built on the basis of the Big Five/FFM. According to our conceptual consideration, the ICD-11 domains cohesively covered the whole palette of personality pathology characteristics within the CPM matrix, reaching from Delta-Minus to Delta-Plus, with Anankastia closing the pattern as being cohesively embedded within the latter. In contrast, the DSM-5 domains revealed a narrower and less neat picture of personality pathology, which was caused basically by the fuzzy position of Psychoticism, but also by a lack of a trait located close to Delta-Plus that serves as the border between normal and pathological personality in the CPM model. Empirical results obtained in our research roughly confirmed these theoretical considerations and predictions.

Psychoticism does not show a specific theoretical position within the CPM model, and therefore its predicted location was deemed to be not justified enough. Not surprising then, in the current research, the congruence indices between the theoretical predictions and empirical location were not satisfactory (i.e., low in Study 1, although acceptable in Study 2). Nevertheless, also the empirical relations between Psychoticism and FFM dimensions turned out to be contradictory to expectations. Namely, Psychoticism did not correlate with Openness, which is supposed to be its counterpart in the normal FFM. Instead, Psychoticism showed significant negative relations with both Conscientiousness and Agreeableness. It is worth noting that such findings are in line with results observed in previous studies (20, 25–28). One explanation proposed, 1 is that Psychoticism is differentially associated with specific various aspects of Openness. In particular, Psychoticism is claimed to be a maladaptive variant of Openness that is positively related to one of its aspects (i.e., perceptual-aesthetic and imaginative) and negatively related to the other one [i.e., intellectual, e.g., (68)]. The results of our research do not support that notion. Our analysis executed on the facet-level traits evidently showed zero correlation between Psychoticism and all of the Openness facets.

In both studies, Psychoticism consistently demonstrated a shift toward Alpha-Minus, which is in accordance with its correlation to FFM dimensions. Our results are then in line with Eysenck's (29) model—relating Psychoticism with antisocial tendencies. However, another explanation can be proposed that seems to be even more justified. According to the non-specificity or heterogeneity of Psychoticism, this phenomenon can be deemed as adhering to another area than personality (disorders). Given the obtained pattern of results and suggestions from previous research, it seems that Psychoticism, which includes perceptual and cognitive dysregulations or even delusional contents (e.g., delusions of thought control), goes far beyond the conceptualization of personality traits, and as a result typically shows a less consistent correlation pattern with them or even does not correlate with personality at all (51). Thus, it seems an important point to recognize that psychotic-like features at large could be better conceptualized in terms of the general level of severity as the degree to which one's capacity for reality testing is compromised, according to the overall degree of PD severity proposed in the ICD-11 diagnostic system (2). Moreover, the absence of Psychoticism in the ICD-11 model is sound with the manner in which schizotypal personality disorder is understood by WHO (i.e., schizotypy is a variant of schizophrenia, rather than a personality disorder). In accord, in the ICD-11, personality disorders form one grouping, whereas schizophrenia, schizotypal, and delusional disorders constitute another^2^. As pointed, rather than seeing Psychoticism as a separate personality trait domain, PD severity may thoroughly encompass and reflect the presence of dissociative states or psychotic-like beliefs or perceptions, and/or highly eccentric behaviors. That approach reflecting core PD features with an emphasis on identifying the severity of personality dysfunction has received great recognition (70–72) and is more consistent with the traditional structural approach to classification of personality organization as neurotic, borderline, and psychotic levels, in which the most severe levels may involve transient psychotic states. Moreover, this also results in avoiding the amount of overlap between levels of personality disturbance (i.e., the impairment in personality functioning) and maladaptive traits [for a discussion, see (11, 55)].

In contrast, the current research suggested the cohesiveness, separateness, and discrimination of the ICD-11 domains in line with previous findings [e.g., (20, 21, 73); for a review, see also (11)]. Data from both our studies revealed sound and cohesive relations of Anankastia with the CPM (perfect fit to the expected location in both studies), as well as FFM domains, indicating that it is a robust and specific dimension of personality pathology. In line with previous works (11, 18, 20, 21), Anankastia showed convergence with the Conscientiousness domain and its facets (i.e., Organization and Responsibility), as well as a strong negative relation with Disinhibition (see Supplementary Material), providing a clear reflection of the conceptual definition of this pathological domain (2). Results were also as good regarding the overall fit of the Anankastia location within the CPM, showing excellent congruence between theoretical expectation and empirical placement. Across both studies, Anankastia showed a very good fit, with no deviation from the expected position (congruence coefficients equal to 1.00). What is more, in accordance with the conceptual perspective of the CPM model, the obtained results suggest that Anankastia should be treated as the opposite pole of the Disinhibition domain.

### Bipolarity as a Consequence of Dimensional Approach—Insight From the Circumplex of Personality Metatraits

Our results concerning the bipolar relations of the Disinhibition vs. Anankastia domains are in line with prior suggestions and previous research strictly investigating the structure of the ICD-11 model (17–21). It is interesting that Tyrer et al. (11) stated that Negative Affectivity is linked with high Neuroticism, Detachment with low Extraversion, Dissociality with low Agreeableness, Disinhibition with low Conscientiousness, and Anankastia with high Conscientiousness. As such, the authors of the ICD-11 trait model do appear to recognize that Anankastia and Disinhibition are opposite to one another, but seem to not make this point explicitly (11, 74). Of note, however, the results on bipolarity do not contradict the presence of distinguishable domains. Based upon the results of analysis within the conceptual framework of the CPM, each of the two specific domains has its own substantive and specific meaning beyond simple opposition. Relatedly, they should be understood as qualitatively distinct entities, which cannot simply be reduced to their opposite characteristics. Essentially, consistent with prior suggestions (17, 73), we assert that it is most suitable (and straightforward for practitioners) to code one to five of the ICD-11 trait domain qualifiers.

However, having three domains being unipolar and the other two domains creating one bipolar dimension in the ICD-11 model might provide an intricacy that one could find a bit confusing. In other words, the ICD-11 model appears to be a five-domain, but only four-factor model. One of the major strengths of the current research is its ability to explain this problem cohesively and shed light on the issue of unipolarity vs. bipolarity of the pathological domains. One can say that the shift from the categorical to the dimensional approach—made by both DSM-5 and ICD-11 assessment system for PD—is an important step in the right direction, albeit a somewhat incomplete one. A fully dimensional approach required bipolar rather than unipolar conceptual thinking and the CPM structure provides such framework for conceptualizing personality pathology.

According to the CPM, each of the ICD-11 domains are unipolar dimensions. However, each of them possess their opposite unipolar dimension, and Anankastia and Disinhibition domains form just a special case of such relations, because in the CPM they are located as the farthest points on the border with healthy personality characteristics, that is, Delta-Plus and Delta-Minus, respectively (see Figures 2, 3). These two metatraits placed into a boundary between healthy and personality disturbance delineate a line of demarcation within the broader model of personality structure that can also enable discrimination between different types of pathology, e.g., in terms of including restraint/constraint vs. impulsivity-relevant features. As a consequence, Anankastia and Disinhibition are pathological domains with some ambiguity or functional potential. Other ICD-11 domains are more unequivocally pathological (as the CPM space below Delta can be seen as an area of personality functioning with specific manifestations of personality disturbance, see Figure 3) and possess healthy, unipolar dimensions as opposites. The opposite pole of Dissociality in the CPM is Stability (in social and motivational areas of functioning), while the opposite pole for Detachment is Plasticity (openness and engagement in novelty and change). Within this framework, Negative Affectivity is the core of personality pathology, with the opposite pole of Integration (well-being), as they are located on bipolar dimension of Gamma. The negative pole of Gamma is labeled Disharmony, as it includes the most maladaptive configuration of personality traits, and its positive pole (Gamma-Plus) is labeled Integration, as it encompasses all functional qualities of personality (including high emotional stability, extraversion, agreeableness, conscientiousness, and openness). Therefore, CPM Gamma reflects general psychopathology vs. general mental health proneness, and is a counterpart of the general factors of: psychopathology (*p* factor), personality disorder (g-PD), or personality [GFP; (9, 10, 43, 45, 46, 75–77)].

The assumption that all personality dysfunctions are represented within a half of the circumplex space that is confined by the Delta-Plus and Delta-Minus was broadly supported by previous studies (9), in particular by Zawadzki's (50) findings on analyses using categorical classification of personality disorders. What is more, hardly available within standard factorial models as the FFM, while inherent to a circumplex framework is the precise determining of the relationships among all distinguished dimensions. Specifically, the CPM encompasses all relations among the ICD-11 dimensions (rather than just between Anankastia vs. Disinhibition), determining the expected pattern of their intercorrelations. For example, Negative Affectivity should be more strongly related with Detachment than with Anankastia, and Detachment should be more strongly related with Anankastia, than with Dissociality, respective to their distance to each-other within the CPM model (see Figure 3 and Supplementary Material). Thus, although the fit of the new ICD-11 dimensional systems of PD to the FFM factorial structure of normal personality is not fully congruent, the CPM model turned out to provide a better framework that justifies distinguished trait-domains as well as their mutual relationships in a cohesive and complete manner.

### Some Measurement and Conceptual Issues. Toward Further Studies

Recently, some efforts also have been made to harmonize the two PD systems, providing the six-domain model of the combined ICD-11 and DSM-5 trait dimensions (32). However, this conjoined model is, for now, a counting algorithm rather than a theoretical proposal, built in line with a suggestion that the two PID-5 scales of Perseveration and Rigid Perfectionism may provide a proxy measure of Anankastia (30–32). On the other hand, recent studies have provided evidence that perhaps only Rigid Perfectionism from those two traits should be used for this purpose (20). Moreover, Bach et al.'s (32) algorithm introduces strict separateness (orthogonality in fact) between Anankastia and Disinhibition, neglecting the emergence of sound scientific evidence that these traits constitute a bipolar dimension [(7, 14, 17–19)]. In our research, the six-domain algorithm showed very poor indices in regards to empirical locations within the personality structure provided by the CPM, with the largest misfit for Anankastia. What is more, unsatisfactory congruence with the conceptually-theoretical assumption was reflected in the distorted pattern of relationships with the FFM traits. The most evident was the strongest correlation of Anankastia with the Neuroticism rather than Conscientiousness domain, and with Depression on the facet level (which correspond with the location of Anankastia near Gamma-Minus). Therefore, both the previous and the current results seem to suggest that the six-domain integrating framework for the ICD-11 and DSM-5 models cannot be seen as final. It is because such integration may require some more profound reconceptualization rather than just reoperationalization or simple reclassification of the personality trait-facets and domains. Moreover, integration may not necessarily be the best solution because one model can simply be superior to another in the conceptualization of PD, as suggested by the presented study.

One more specific point is also worth recognizing. Namely, although Anankastia revealed its clear conceptual and empirical superiority over Psychoticism in terms of fit to the CPM personality structure, and empirically the DSM-5 domains cover only a half area of personality pathology (restricted between Gamma-Minus to Delta-Minus), the empirical congruencies of the overall ICD-11 model were only slightly better than in the case of the DSM-5 model. This was due to some “weak points” present in both models, i.e., domains that revealed the lowest fit. These domains are Disinhibition in the ICD-11 model and Detachment in DSM-5. Problems revealed by these domains could possibly be caused by some measurement imperfections, i.e., properties of PiCD and PID-5 inventories. However, there can also be some conceptual reasons that play a significant role in this context. Taking the CPM framework into account it becomes evident that Disinhibition's pathological domain—as located in Delta-Minus rather than Alpha-Minus—should not include purely antisocial tendencies, primarily motivated by harmful or aggressive impulses. Instead, it should possess excessive sensation seeking related with risk taking which seems to be underrepresented within the ICD-11 model (2), whereas it is present in DSM-5 (1). In turn, all antisocial tendencies should be grouped within the Dissociality/Antagonism domain, however, its characteristics related to self-centeredness, grandiosity, attention-seeking, and expectation of others' admiration should be strongly saturated by vulnerability, sense of entitlement, exploitativeness of others, rivalry and hostility. In other words, it seems that narcissistic characteristics included in Dissociality/Antagonism should possess slightly more aspects of vulnerable narcissism and narcissistic rivalry and slightly fewer aspects of grandiose narcissism and narcissistic admiration (56, 78). It became of crucial importance when the limited number of facets (e.g., three) for the domain are selected (1, 32).

Regarding the Detachment domain in the DSM-5 model, it simply should not possess tendencies to experience endogenic anxiety-based and hostile negative emotions, such as depressivity and distrust (suspiciousness). On the other hand, the selection of three out of six facets for the Detachment domain (i.e., withdrawal, intimacy avoidance, and anhedonia) introduced by APA (1) for diagnostic purposes, seems to be a promising proposal, as these three are important and basically pure elements of the domain. However, in light of the CPM framework is seems worthwhile to consider if social avoidance and distance should not be supplemented by shyness as well as submissiveness and dependency in close relationships, despite a generally small number and unwillingness for establishing the latter.

Summing up, given the conceptually sound structure of the ICD-11 trait domains and their theoretically meaningful and in general empirically confirmed pattern of relations with the CPM integrative model of whole personality structure, the ICD-11 PD model seems to surpass the DSM-5 proposal. However, it should be noted that both PD models' emphasis on personality processes and dimensionality, which contrasts with the old descriptive approach using polythetic-categorical concepts, has been well-recognized (55). What is more, both proposals should not be reduced to just the Pathological Big Five models. It is true especially for the DSM-5 which is a complex diagnostic system with also other very important elements that are unique in comparison with the ICD-11 model. Firstly, the DSM-5 contains an empirically derived catalog of 25 trait-facets of the Pathological Big Five as well as empirically confirmed model of personality functioning included in Criterion A (8). Secondly—and also in contrast to the ICD-11 model—the combination of Criterion A (disturbances in both self and interpersonal functioning) and Criterion B (maladaptive personality trait-facets) of the DSM-5 system allows to generate a hybrid categorical-dimensional diagnosis (1). Prior research has shown support for the 2-fold structure of Criterion A and its at least partial distinctiveness from the five dimensions of Criterion B (55, 79). Notably, the AMPD allows reflection on multiple personality constructs and paradigms, embracing psychodynamic, interpersonal, personological paradigms (embedded within Criterion A), and also multivariate and empirical paradigms (reflected mostly within Criterion B) to PD diagnosis (80, 81). By requiring both Criteria A and B for PD diagnosis, the DSM-5 system is reasonably theoretically comprehensive, with a pluralistic perspective. It itself requires only that a broad spectrum of constructs and paradigms be considered—but no theoretical paradigm in the DSM-5 system is favored. A clinician has the opportunity to operate from his/her own conceptual point of view. This confers scientific and practical advantages that deserve more empirical articulation from researchers in the field than they have received so far. Therefore, the DSM-5 contribution and advance within PD nosology, goes beyond the dimensionalization of diagnosis. The same is roughly true for ICD-11, so future research should also show if the latter can take some benefits from the DSM-5 system, in particular its conceptualization of psychological functioning (Criterion A) or lower-order traits (catalog of facets of Pathological Big Five).

## Limitations

Our study is not free of limitations. Although the current analyses utilized two adequately large samples with adequate score variance for the measures, our findings should be replicated and extended to other samples exhibiting a broad range of clinical symptomatology. Moreover, one should keep in mind that the data are based exclusively on self-report measures, which is different from a clinical rating made by professionals. It would be distinctly useful to replicate and extend the current findings using both informant and self-reports. Finally, further methodological research, including simulations, could also answer the question whether a different cutoff for RMSEA in case of circumplex models is justified and what exactly this cutoff should be.

## Conclusions

Our research offers a direct empirical comparison of two catalogs of pathological personality trait domains derived from the ICD-11 and DSM−5 Section III AMPD diagnostic system, providing a step forward for the validation of both PD models. In respect to the current debate on the overall expected four- vs. five-factor models of personality pathology, we used the circumplex matrix delineating theoretically meaningful predicted locations of constructs to provide an evaluation and a comprehensive comparison of the ICD-11 and DSM−5 models. Building upon the CPM structure, the findings generally support that the domain of Anankastia may provide a more robust and specific domain of personality pathology, as more cohesively located within the overall structure of personality, than Psychoticism. Additionally, in line with previous research (17, 18, 20), the results corroborated the bipolar structure of the Disinhibition vs. Anankastia domains. Importantly, the notion that the traits can be plainly portrayed as a bipolar dimension cannot be seen as the reduction of these domains simply to their opposite characteristics. As shown, each of the traits has its own substantive and specific meaning beyond simple opposition. These conclusions were roughly confirmed from the perspective of the FFM results, which we also included in our study as the, to date, most often used context of normal personality structure.

In general, our research yielded a new view as provided from the circumplex perspective on personality structure to progress the debate on potential avenues for the integration of the ICD-11 and DSM-5 trait models (in line with strong similarities in the models) and speculated the superiority of one of them (in accord with some important conceptually meaningful differences). Obviously, we adopted a scientifically essential, however, not only important structural (or structuralist) perspective provided by the CPM model. Nevertheless, of crucial importance, further examination of how clinician-reported ICD-11 and DSM-5 traits are empirically organized may somewhat highlight aspects of their validity and utility for clinical practice. Ultimately, advantages for clinical practice should be decisive, but, in order for the latter to be retained, a scientific and theoretical cohesiveness seems necessary and the CPM can provide a useful perspective for achieving such purposes as we have shown in our study.

## Data Availability Statement

The datasets presented in this study can be found in online repository at Open Science Framework (Center for Open Science): https://osf.io/7pw3q/ Dimensional Models of Personality Pathology project: doi: https://doi.org/10.17605/OSF.IO/7PW3Q

## Ethics Statement

The studies involving human participants were reviewed and approved by the Commission of Ethics and Bioethics at the Cardinal Stefan Wyszyński University in Warsaw. Written informed consent for participation was not required for this study in accordance with the national legislation and the institutional requirements.

## Author Contributions

WS and JC designed and conceptualized the research and revised the paper and improved it to the final version. WS collected the data and prepared the dataset. WS and PŁ ran the statistical analyses. PŁ prepared the literature review and wrote the first version of the manuscript. All authors contributed to the article and approved the submitted version.

## Conflict of Interest

The authors declare that the research was conducted in the absence of any commercial or financial relationships that could be construed as a potential conflict of interest.

## Publisher's Note

All claims expressed in this article are solely those of the authors and do not necessarily represent those of their affiliated organizations, or those of the publisher, the editors and the reviewers. Any product that may be evaluated in this article, or claim that may be made by its manufacturer, is not guaranteed or endorsed by the publisher.

## Footnotes

^1^Other variables measured in the samples that were not utilized in the current analyses were beyond the scope of the presented paper.

^2^An important point to recognize is that a comparable proposal was actually considered for the DSM-5, in which schizotypal personality disorder would be shifted out of the PD section and into the schizophrenia section (69), however, the proposal was finally rejected.

## References

[1] American Psychiatric Association. Diagnostic and Statistical Manual of Mental Disorders. 5th ed. Washington, DC: American Psychiatric Association (2013). p. 729. 10.1176/appi.books.9780890425596

[2] World Health Organization. ICD-11, the 11th Revision of the International Classification of Diseases. (2020). Available online at: https://icd.who.int/en (accessed December 31, 2020).

[3] Clark LA. Resolving taxonomic issues in personality disorders. J Pers Disord. (1992) 6:360–76.

[4] Clark LA Cuthbert B Lewis-Fernández R Narrow WE Reed GM. Three approaches to understanding and classifying mental disorder: ICD-11, DSM-5, and the National Institute of Mental Health's Research Domain Criteria (RDoC). Psychol Sci Public Interest. (2017) 18:72–145. 10.1177/1529100617727266 29211974

[5] Krueger RF. Personality disorders are the vanguard of the post-DSM−5.0 era. Personal Disord. (2013) 4:355–62. 10.1037/per0000028 24378165

[6] Widiger TA Clark LA. Toward DSM-V and the classification of psychopathology. Psychol Bull. (2000) 126:946–63. 10.1037/0033-2909.126.6.946 11107884

[7] Widiger TA Simonsen E. Alternative dimensional models of personality disorder: finding a common ground. J Pers Disord. (2005) 19:110–30. 10.1521/pedi.19.2.110.62628 15899712

[8] Krueger RF Markon KE. The role of the DSM−5 personality trait model in moving toward a quantitative and empirically based approach to classifying personality and psychopathology. Annu Rev Clin Psychol. (2014) 10:477–501. 24329179 10.1146/annurev-clinpsy-032813-153732

[9] Strus W Cieciuch J. Towards a synthesis of personality, temperament, mo- tivation, emotion and mental health models within the Circumplex of Personality Metatraits. J Res Pers. (2017) 66:70–95. 10.1016/j.jrp.2016.12.002

[10] Strus W Cieciuch J. The Circumplex of Personality Metatraits and the HEXACO model: towards refinement and integration. J Personal. (2021) 89:803–18. 10.1111/jopy.12616 33421127

[11] Tyrer P Mulder R Kim YR Crawford MJ. The Development of the ICD-11 classification of personality disorders: an amalgam of science, pragmatism, and politics. Ann Rev Clin Psychol. (2019) 15:481–502. 10.1146/annurev-clinpsy-050718-095736 30601688

[12] Widiger TA McCabe GA. The alternative model of personality disorders (AMPD) from the perspective of the five-factor model. Psychopathology. (2020) 53:149–56. 10.1159/000507378 32526758

[13] Mulder RT Horwood J Tyrer P Carter J Joyce PR. Validating the proposed ICD-11 domains. Personal Ment Health. (2016) 10:84–95. 10.1002/pmh.1336 27120419

[14] Skodol AE. Personality disorders in DSM−5. Annu Rev Clin Psychol. (2012) 8:317–44. 10.1146/annurev-clinpsy-032511-143131 22458868

[15] Krueger RF Derringer J Markon KE Watson D Skodol AE. Initial construction of a maladaptive personality trait model and inventory for DSM−5. Psychol Med. (2012) 42:1879–90. 10.1017/S0033291711002674 22153017 PMC3413381

[16] Volkert J Gablonski TC Rabung S. Prevalence of personality disorders in the general adult population in Western countries: systematic review and meta-analysis. Br J Psychiatr. (2018) 213:709–15. 10.1192/bjp.2018.202 30261937

[17] Oltmanns JR Widiger TA. A self-report measure for the ICD-11 dimensional trait model proposal: the Personality Inventory for ICD-11. Psychol Assess. (2018) 30:154–69. 10.1037/pas0000459 28230410 PMC5930359

[18] Oltmanns JR Widiger TA. Evaluating the assessment of the ICD-11 personality disorder diagnostic system. Psychol Assess. (2019) 31:674–84. 10.1037/pas0000693 30628821 PMC6488396

[19] Oltmanns JR Widiger TA. The five-factor personality inventory for ICD-11: a facet-level assessment of the ICD-11 trait model. Psychol Assess. (2019) 2019:31234. 10.31234/osf.io/ycgwn 31414852 PMC6928398

[20] McCabe GA Widiger TA. A comprehensive comparison of the ICD-11 and DSM−5 section III personality disorder models. Psychol Assess. (2020) 32:72–84. 10.1037/pas0000772 31580095

[21] Carnovale M Sellbom M Bagby RM. The Personality Inventory for ICD-11: investigating reliability, structural and concurrent validity, and method variance. Psychol Assess. (2020) 32:8–17. 10.1037/pas0000776 31556679

[22] Al-Dajani N Gralnick TM Bagby RM. A psychometric review of the Personality Inventory for DSM−5 (PID−5): current status and future directions. J Pers Assess. (2016) 98:62–s81. 10.1080/00223891.2015.1107572 26619968

[23] Bastiaens T Smits D De Hert M Vanwalleghem D Claes L. DSM−5 Section III personality traits and Section II personality disorders in a Flemish community sample. Psychiatry Res. (2016) 238:290–8. 10.1016/j.psychres.2016.02.056 27086247

[24] Crego C Gore WL Rojas SL Widiger TA. The discriminant (and convergent) validity of the Personality Inventory for. DSM−5. Personality disorders: theory. Res Treat. (2015) 6:321–35. 10.1037/per0000118 25894855

[25] Gore WL Widiger TA. The DSM−5 dimensional trait model and five-factor models of general personality. J Abnorm Psychol. (2013) 122:816–21. 10.1037/a0032822 23815395

[26] Quilty LC Ayearst L Chmielewski M Pollock BG Bagby RM. The psychometric properties of the personality inventory for DSM−5 in an APA DSM−5 field trial sample. Assessment. (2013) 20:362–9. 10.1177/1073191113486183 23588687

[27] Watson D Stasik SM Ro E Clark LA. Integrating normal and pathological personality: relating the DSM−5 traitdimensional model to general traits of personality. Assessment. (2013) 20:312–26. 10.1177/1073191113485810 23596272

[28] Watters CA Bagby RM Sellbom M. Meta-analysis to derive an empirically based set of personality facet criteria for the alternative DSM-5 model for personality disorders. Personal Disord. (2019) 10:97–104. 10.1037/per0000307 30520649

[29] Eysenck HJ Eysenck SBG. Psychoticism as a Dimension of Personality. New York, NY: Crane, Russak & Co. (1976).

[30] Bach B Sellbom M Kongerslev M Simonsen E Krueger RF Mulder R. Deriving ICD-11 personality disorder domains from DSM−5 traits: initial attempt to harmonize two diagnostic systems. Acta Psychiatr Scand. (2017) 136:108–17. 10.1111/acps.12748 28504853

[31] Bach B Sellbom M Skjernov M Simonsen E. ICD-11 and DSM−5 personality trait domains capture categorical personality disorders: finding a common ground. Austr N Zeal J Psychiatr. (2018) 52:425–34. 10.1177/0004867417727867 28835108

[32] Bach B Kerber A Aluja A Bastiaens T Keeley JW Claes L. International assessment of DSM-5 and ICD-11 personality disorder traits: toward a common nosology in DSM-5.1. Psychopathology. (2020) 53:179–88. 10.1159/000507589 32369820

[33] Ashton MC Lee K. Empirical, theoretical, and practical advantages of the HEXACO model of personality structure. Pers Soc Psychol Rev. (2007) 11:150–66. 10.1177/1088868306294907 18453460

[34] Digman JM. Higher-order factor of the Big Five. J Pers Soc Psychol. (1997) 73:1246–56. 10.1037/0022-3514.73.6.1246 9418278

[35] DeYoung CG Peterson JB Higgins DM. Higher-order factors of the Big Five predict conformity: are there neuroses of health? Personal Individ Diff. (2002) 33:533–52. 10.1016/S0191-8869(01)00171-4

[36] Cieciuch J Strus W. The two-factor model of personality. In: Zeigler-Hill V Shackelford TK, editors, Encyclopedia of Personality and Individual Differences. Cham: Springer International Publishing AG (2020). p. 5599–615. 10.1007/978-3-319-28099-8_2129-1

[37] Ashton MC Lee K De Vries RE. The HEXACO honesty-humility, agreeableness, and emotionality factors: a review of research and theory. Personal Soc Psychol Rev. (2014) 18:139–52. 10.1177/1088868314523838 24577101

[38] Saucier G. Recurrent personality dimensions in inclusive lexical studies: indications for a big six structure. J Pers. (2009) 77:1577–614. 10.1111/j.1467-6494.2009.00593.x 19678873

[39] Thalmayer AG Saucier G. The questionnaire Big Six in 26 nations: developing cross-culturally applicable Big Six, Big Five and Big Two Inventories. Eur J Pers. (2014) 28:482–96. 10.1002/per.1969

[40] Markon KE Krueger RF Watson D. Delineating the structure of normal and abnormal personality: an integrative hierarchical approach. J Pers Soc Psychol. (2005) 88:139–57. 10.1037/0022-3514.88.1.139 15631580 PMC2242353

[41] Wright AGC Thomas KM Hopwood CJ Markon KE Pincus AL Krueger RF. The hierarchical structure of DSM-5 pathological personality traits. J Abnorm Psychol. (2012) 121:951–7. 10.1037/a0027669 22448740 PMC3389150

[42] Strus W Cieciuch J Rowiński T. The circumplex of personality metatraits: a synthesizing model of personality based on the Big Five. Rev Gen Psychol. (2014) 18:273–86. 10.1037/gpr0000017

[43] Strus W Cieciuch J. Are the questionnaire and the psycho-lexical Big Twos the same? Towards an integration of personality structure within the Circumplex of Personality Metatraits. Int J Personal Psychol. (2019) 5:18–35. 10.21827/ijpp.5.35594

[44] Widiger TA Oltmanns JR. Neuroticism is a fundamental domain of personality with enormous public health implications. World Psychiatr Off J World Psychiatr Assoc. (2017) 16:144–5. 10.1002/wps.20411 28498583 PMC5428182

[45] Oltmanns JR Smith GT Oltmanns TF Widiger TA. General factors of psychopathology, personality, and personality disorder: across domain comparisons. Clin Psychol Sci. (2018) 6:581–9. 10.1177/2167702617750150 30221082 PMC6132273

[46] Smith GT Atkinson EA Davis HA Riley EN Oltmanns JR. The general factor of psychopathology. Ann Rev Clin Psychol. (2020) 16:75–98. 10.1146/annurev-clinpsy-071119-115848 32040926

[47] Brud PP Rogoza R Cieciuch J. Personality underpinnings of dark personalities: an example of Dark Triad and deadly sins. Pers Individ Dif. (2020) 163:110085. 10.1016/j.paid.2020.110085

[48] Rogoza R Kowalski CM Schermer JA. Dark Triad traits within the framework of the Circumplex Model of Personality Metatraits. J Individ Diff. (2019) 40:168–76. 10.1027/1614-0001/a000289

[49] Rogoza R Cieciuch J Strus W. A three-step procedure for analysis of circumplex models: an example of narcissism located within the circumplex of personality metatraits. Pers Individ Dif. (2021) 169:109775. 10.1016/j.paid.2019.109775

[50] Zawadzki B. The location of personality disorders in the Circumplex of Personality Metatraits. Ann Psychol. (2017) 20:493–512. 10.18290/rpsych.2017.20.2-7en

[51] Widiger TA Crego C. HiTOP thought disorder, DSM−5 psychoticism, and five-factor model openness. J Res Pers. (2019) 80:72–7. 10.1016/j.jrp.2019.04.008

[52] Cieciuch J Łakuta P Strus W Oltmanns JR Thomas Widiger T. Assessment of personality disorder in the ICD-11 diagnostic system: polish validation of the Personality Inventory for ICD-11. Psychiatria Polska. (2021) 2021:138563. 10.12740/PP/OnlineFirst/138563 37098193

[53] Rowiński T Kowalska-Dabrowska M Strus W Cieciuch J Czuma I Zechowski C. Measurement of pathological personality traits according to the DSM-5: a Polish adaptation of the PID-5. Part II – empirical results. Psychiatria Polska. (2019) 53:23–48. 10.12740/PP/OnlineFirst/86478 31008463

[54] Thomas KM Yalch MM Krueger RF Wright AG Markon KE Hopwood CJ. The convergent structure of DSM-5 personality trait facets and five-factor model trait domains. Assessment. (2013) 20:308–11. 10.1177/1073191112457589 22946103

[55] Zimmermann J Kerber A Rek K Hopwood CJ Krueger RF. A brief but comprehensive review of research on the Alternative DSM-5 model for personality disorders. Curr Psychiatry Rep. (2019) 21:92. 10.1007/s11920-019-1079-z 31410586

[56] Rogoza R Cieciuch J Strus W Baran T. Seeking a common framework for research on narcissism: an attempt to integrate the different faces of narcissism within the Circumplex of Personality Metatraits. Eur J Pers. (2019) 33:437–55. 10.1002/per.2206

[57] Soto CJ John OP. The next Big Five Inventory (BFI-2): Developing and assessing a hierarchical model with 15 facets to enhance bandwidth, fidelity, and predictive power. J Pers Soc Psychol. (2017) 113:117–43. 10.1037/pspp0000096 27055049

[58] John OP Naumann LP Soto CJ. Paradigm shift to the integrative Big Five trait taxonomy: history, measurement, and conceptual issues. In: John OP Robins RW Pervin LA editors, Handbook of Personality: Theory and Research. 3rd ed. New York, NY: Guilford (2008). p. 114–58.

[59] Grassi M Luccio R Di Blas L. CircE: an R implementation of Browne's circular stochastic process model. Behav Res Methods. (2010) 42:55–73. 10.3758/BRM.42.1.55 20160286

[60] Zimmerman J Wright AG. Beyond description in interpersonal construct validation: methodological advances in the circumplex structural summary approach. Assessment. (2017) 24:3–23. 10.1177/1073191115621795 26685192

[61] Barrett P. Orthosim: Target-Comparison Matrix Fitting. (2013). Available online at: www.pbarrett.net (accessed December 31, 2020).

[62] Byrne BM. Structural Equation Modeling With EQS and EQS/Windows: Basic Concepts, Applications, and Programming. Thousand Oaks: Sage Publications (1994).

[63] Schermelleh-Engel K Moosbrugger H Müller H. Evaluating the fit of structural equation models: tests of significance and descriptive goodness-of-fit measures. Methods Psychol Res. (2003) 8:23–74.

[64] Pincus AL Gurtman MB. Interpersonal assessment. In: Wiggins JS editor, Paradigms of Personality Assessment. New York, NY: Guilford (2003). p. 246–61.

[65] DeYoung CG Weisberg YJ Quilty LC Peterson JB. Unifying the aspects of the Big Five, the interpersonal circumplex, and trait affiliation. J Pers. (2013) 81:465–75. 10.1111/jopy.12020 23126539

[66] McCrae RR Zonderman AB Bond MH Costa PT Paunonen SV. Evaluating replicability of factors in the Revised NEO Personality Inventory: confirmatory factor analysis versus Procrustes rotation. J Pers Soc Psychol. (1996) 70:552–66. 10.1037/0022-3514.70.3.552

[67] Terracciano A McCrae RR Hagemann D Costa PT. Individual difference variables, affective differentiation, and the structures of affect. J Pers. (2003) 71:669–703. 10.1111/1467-6494.7105001 12932207 PMC2580756

[68] DeYoung CG Carey BE Krueger RF Ross SR. Ten aspects of the Big Five in the personality inventory for DSM-5. Personal Disord. (2016) 7:113–23. 10.1037/per0000170 27032017 PMC4818974

[69] First MB Bell CB Cuthbert B Krystal JH Malison R Offord DR. Personality disorders and relational disorders: a research agenda for addressing crucial gaps in DSM. In: Kupfer DJ First MB & Regier DA editors, A Research Agenda for DSM-V. Washington, DC: American Psychiatric Association (2002). p. 123–99.

[70] Sharp C Wright AG Fowler JC Frueh BC Allen JG Oldham J. The structure of personality pathology: both general ('g') and specific ('s') factors? J Abnorm Psychol. (2015) 124:387–98. 10.1037/abn0000033 25730515

[71] Williams TF Scalco MD Simms LJ. The construct validity of general and specific dimensions of personality pathology. Psychol Med. (2018) 48:834–48. 10.1017/S0033291717002227 28826417

[72] Clark LA Nuzum H Ro E. Manifestations of personality impairment severity: comorbidity, course/prognosis, psychosocial dysfunction, and 'borderline' personality features. Curr Opin Psychol. (2018) 21:117–21. 10.1016/j.copsyc.2017.12.004 29291458

[73] Bach B Christensen S Kongerslev MT Sellbom M Simonsen E. Structure of clinician-reported ICD-11 personality disorder trait qualifiers. Psychol Assess. (2020) 32:50–9. 10.1037/pas0000747 31328934

[74] Reed GM First MB Kogan CS Hyman SE Gureje O Gaebel W. Innovations and changes in the ICD-11 classification of mental, behavioural and neurodevelopmental disorders. World Psychiatr. (2019) 18:3–19. 10.1002/wps.20611 30600616 PMC6313247

[75] Becker P. A multifacets circumplex model of personality as a basis for the description and therapy of personality disorders. J Pers Disord. (1998) 12:213–25. 10.1521/pedi.1998.12.3.213 9785264

[76] Musek J. A general factor of personality: evidence of the big one in the five-factor model. J Res Pers. (2007) 41:1213–33. 10.1016/j.jrp.2007.02.003

[77] Rushton JP Irving P. The general factor of personality: normal and abnormal. In: Chamorro-Premuzic T von Stumm S Furnham A editors, The Wiley-Blackwell Handbook Of Individual Differences. London: Blackwell Publishing Ltd. (2011). p. 134–63.

[78] Back MD Küfner ACP Dufner M Gerlach TM Rauthmann JF Denissen JJA. Narcissistic admiration and rivalry: disentangling the bright and dark sides of narcissism. J Pers Soc Psychol. (2013) 105:1013–37. 10.1037/a0034431 24128186

[79] Zimmermann J Böhnke JR Eschstruth R Mathews A Wenzel K Leising D. The latent structure of personality functioning: investigating criterion a from the alternative model for personality disorders in DSM-5. J Abnorm Psychol. (2015) 124:532–48. 10.1037/abn0000059 26052618

[80] Mulay AL Cain NM Waugh MH Hopwood CJ Adler JM Garcia DJ. Personality constructs and paradigms in the alternative DSM-5 model of personality disorder. J Pers Assess. (2018) 100:593–602. 10.1080/00223891.2018.1477787 29902081

[81] Waugh MH Hopwood CJ Krueger RF Morey LC Pincus AL Wright A. Psychological asessment with the DSM-5 Alternative Model for personality disorders: tradition and innovation. Prof Psychol Res Pr. (2017) 48:79–89. 10.1037/pro0000071 28450760 PMC5403154

