# Supplementary material for: Anankastia or Psychoticism? Which One Is Better Suited for the Fifth Trait in the Pathological Big Five: Insight From the Circumplex of Personality Metatraits Perspective
Source: Front Psychiatry. 2021 Oct 14;12:648386. doi: 10.3389/fpsyt.2021.648386 (PMC8551367; doi:10.3389/fpsyt.2021.648386)
Supplement: Supplementary file 1 [file Table_1.docx]

Supplementary Material

**Anankastia or Psychoticism? Which one is better suited for the fifth trait in the Pathological Big Five: Insight from the Circumplex of Personality Metatraits perspective**

**Włodzimierz Strus^*^, Patryk Łakuta, Jan Cieciuch**

*** Correspondence: Corresponding Author: w.strus@uksw.edu.pl**

**Table A.** Descriptive statistics, reliability estimates, and correlations of trait-dimensions for Study 1 (below diagonal) and Study 2 (above diagonal).

|  |  | CPM | | | | | | | | ICD-11 | | | | | DSM-5 | | | | | Six-domain model | | | | | | *M* (*SD*) | ⍺ |
| --- | --- | --- | --- | --- | --- | --- | --- | --- | --- | --- | --- | --- | --- | --- | --- | --- | --- | --- | --- | --- | --- | --- | --- | --- | --- | --- | --- |
|  | Trait | Delta Plus | Alpha Plus | Gamma Plus | Beta Plus | Delta Minus | Alpha Minus | Gamma Minus | Beta Minus | NA | DT | DL | DN | AK | NA | DT | AN | DN | PS | NA | DT | AN | DN | AK | PS |  |  |
| CPM | Delta-Plus | - | .44 | .14 | -.22 | -.49 | -.36 | -.11 | .46 | -.18 | .27 | -.17 | -.46 | .52 | -.15 | .08 | -.25 | -.46 | -.21 | -.17 | .15 | -.19 | -.28 | .13 | -.14 | 3.22 (0.53); 3.24 (0.54) | .72/.75 |
|  | Alpha-Plus | .43 | - | .56 | .29 | -.23 | -.49 | -.46 | -.16 | -.33 | -.17 | -.29 | -.48 | .33 | -.32 | -.28 | -.33 | -.38 | -.20 | -.17 | -.17 | -.27 | -.36 | .08 | -.12 | 3.78 (0.46); 3.74 (0.46) | .69/.70 |
|  | Gamma-Plus | -.02 | .51 | - | .51 | .05 | -.40 | -.71 | -.40 | -.48 | -.41 | -.17 | -.25 | .06 | -.43 | -.57 | -.19 | -.14 | -.18 | -.22 | -.35 | -.12 | -.21 | -.02 | -.09 | 3.89 (0.53); 3.83 (0.52) | .80/.80 |
|  | Beta-Plus | -.23 | .24 | .60 | - | .41 | -.05 | -.37 | -.64 | -.14 | -.41 | .08 | -.02 | -.04 | -.09 | -.37 | .11 | .13 | .14 | .02 | -.35 | .11 | .01 | .03 | .18 | 3.64 (0.58); 3.55 (0.58) | .81/.81 |
|  | Delta-Minus | -.44 | -.19 | .17 | .45 | - | .51 | .15 | -.36 | .17 | -.16 | .38 | .46 | -.33 | .22 | .02 | .42 | .60 | .33 | .16 | -.04 | .34 | .45 | .06 | .29 | 2.74 (0.66); 2.82 (0.69) | .80/.82 |
|  | Alpha-Minus | -.31 | -.50 | -.32 | -.04 | .51 | - | .54 | .04 | .48 | .10 | .57 | .47 | -.16 | .47 | .35 | .57 | .39 | .35 | .32 | .23 | .43 | .47 | .17 | .30 | 2.15 (0.65); 2.24 (0.66) | .81/.83 |
|  | Gamma-Minus | .05 | -.40 | -.67 | -.46 | .02 | .38 | - | .41 | .63 | .43 | .22 | .36 | -.03 | .61 | .68 | .30 | .24 | .36 | .41 | .48 | .22 | .38 | .11 | .28 | 2.31 (0.73); 2.47 (0.75) | .86/.87 |
|  | Beta-Minus | .50 | -.10 | -.49 | -.63 | -.34 | .12 | .44 | - | .20 | .47 | -.08 | -.06 | .22 | .20 | .44 | -.09 | -.18 | -.08 | .09 | .41 | -.08 | -.02 | .10 | -.10 | 2.44 (0.60); 2.48 (0.56) | .77/.75 |
| ICD-11 | Negative Affect | -.02 | -.26 | -.38 | -.29 | .09 | .32 | .63 | .26 | - | .17 | .24 | .38 | .08 | .78 | .50 | .35 | .24 | .46 | .70 | .32 | .24 | .49 | .27 | .39 | 2.75 (0.66); 2.81 (0.65) | .88/.86 |
|  | Detachment | .25 | -.13 | -.44 | -.45 | -.20 | .06 | .47 | .49 | .28 | - | .14 | .14 | .17 | .16 | .68 | .05 | -.04 | .17 | -.03 | .64 | .01 | .04 | .10 | .13 | 2.18 (0.59); 2.21 (0.61) | .85/.84 |
|  | Dissociality | -.15 | -.26 | -.02 | .23 | .41 | .51 | .13 | -.05 | .01 | .19 | - | .43 | -.05 | .27 | .28 | .73 | .32 | .38 | .12 | .27 | .61 | .35 | .26 | .33 | 2.05 (0.54); 2.07 (0.56) | .80/.81 |
|  | Disinhibition | -.36 | -.48 | -.09 | -.01 | .41 | .54 | .26 | -.01 | .36 | .20 | .36 | - | -.53 | .41 | .28 | .41 | .76 | .44 | .27 | .18 | .32 | .70 | -.18 | .32 | 2.11 (0.54); 2.20 (0.59) | .83/.84 |
|  | Anankastia | .44 | .26 | -.09 | -.05 | -.29 | -.22 | .15 | .22 | .23 | .17 | -.05 | -.37 | - | .11 | .13 | -.02 | .64 | -.04 | .13 | .17 | .01 | -.29 | .48 | -.01 | 3.30 (0.44); 3.24 (0.51) | .72/.79 |
| DSM-5 | Negative Affect | .07 | -.24 | -.37 | -.21 | .16 | .43 | .67 | .30 | .74 | .29 | .21 | .35 | .20 | - | .63 | .54 | .31 | .63 | .84 | .39 | .43 | .56 | .37 | .53 | 1.06 (0.37); 1.13 (0.38) | .94/.95 |
|  | Detachment | .19 | -.21 | -.50 | -.36 | -.06 | .24 | .72 | .44 | .50 | .69 | .24 | .22 | .19 | .69 | - | .41 | .15 | .48 | .36 | .84 | .33 | .34 | .29 | .38 | 0.69 (0.40); 0.76 (0.41) | .94/.94 |
|  | Antagonism | -.16 | -.26 | -.04 | .20 | .46 | .61 | .20 | -.05 | .19 | .06 | .73 | .42 | -.07 | .48 | .33 | - | .37 | .57 | .38 | .35 | .88 | .49 | .37 | .49 | 0.73 (0.43); 0.77 (0.44) | .95/.95 |
|  | Disinhibition | -.41 | -.34 | .03 | .15 | .58 | .42 | .09 | -.19 | .05 | .01 | .32 | .60 | -.51 | .14 | .12 | .44 | - | .42 | .20 | .09 | .28 | .74 | -.32 | .32 | 1.18 (0.33); 1.22 (0.35) | .87/.90 |
|  | Psychoticism | -.05 | -.22 | -.12 | .03 | .25 | .43 | .41 | .14 | .38 | .23 | .43 | .41 | .05 | .60 | .55 | .55 | .32 | - | .50 | .36 | .49 | .57 | .31 | .91 | 0.60 (0.46); 0.76 (0.49) | .93/.94 |
| Six-domain model | Negative Affect | .07 | -.21 | -.32 | -.20 | .07 | .30 | .56 | .25 | .69 | .10 | .03 | .23 | .23 | .85 | .46 | .30 | .00 | .39 | - | .19 | .32 | .42 | .30 | .43 | 2.23 (1.24); 2.30 (1.29) | .76/.76 |
|  | Detachment | .22 | -.19 | -.43 | -.38 | -.10 | .18 | .53 | .44 | .32 | .61 | .19 | .11 | .13 | .43 | .83 | .27 | .09 | .39 | .27 | - | .31 | .23 | .26 | .28 | 1.36 (0.98); 1.39 (0.95) | .66/.65 |
|  | Antagonism | -.13 | -.22 | -.04 | .16 | .35 | .48 | .16 | -.03 | .09 | .10 | .64 | .34 | -.06 | .34 | .28 | .88 | .35 | .52 | .20 | .25 | - | .39 | .33 | .41 | 1.69 (1.18); 1.69 (1.14) | .80/.77 |
|  | Disinhibition | -.22 | -.37 | -.07 | .01 | .42 | .42 | .29 | .01 | .36 | .13 | .29 | .63 | -.24 | .46 | .32 | .45 | .70 | .48 | .35 | .24 | .32 | - | .07 | .47 | 1.79 (1.10); 1.89 (1.15) | .71/.75 |
|  | Anankastia | .24 | .02 | -.21 | -.07 | .04 | .22 | .37 | .25 | .37 | .21 | .24 | -.04 | .42 | .55 | .40 | .33 | -.31 | .37 | .47 | .31 | .27 | .10 | - | .35 | 2.08 (1.20); 2.10 (1.12) | .76/.75 |
|  | Psychoticism | -.07 | -.16 | -.05 | .08 | .24 | .35 | .29 | .08 | .28 | .17 | .42 | .37 | -.01 | .48 | .44 | .51 | .32 | .91 | .30 | .31 | .49 | .45 | .30 | - | 1.13 (1.03); 1.51 (1.10) | .73/.74 |

*Note*. NA – Negative Affectivity, DT – Detachment, DN – Disinhibition, DL – Dissociality, AN – Antagonism, AK – Anankastia; PS – Psychoticism. Mean, standard deviation, and reliability estimates (i.e., Cronbach’s ⍺ coefficients) are presented for Study 1/Study 2, accordingly. In Study 1 (*N* = 242), correlations greater than |.12| are significant at *p* < .05 (two-tailed). In Study 2 (*N* = 355), correlations greater than |.10| are significant at *p* < .05 (two-tailed).

**Table B.** Descriptive statistics, reliability estimates, and correlations of FFM domains and facets with CPM metatraits in Study 1 (*N* = 242).

|  | Trait | Delta Plus | Alpha Plus | Gamma Plus | Beta Plus | Delta Minus | Alpha Minus | Gamma Minus | Beta Minus | *M* (*SD*) | ⍺ |
| --- | --- | --- | --- | --- | --- | --- | --- | --- | --- | --- | --- |
| FFM domains | Neuroticism | -.10 | -.37 | -52 | -.33 | .04 | .37 | .72 | .24 | 2.77 (.74) | .90 |
|  | Extraversion | -.32 | .18 | .57 | .68 | .42 | .04 | -.58 | -.58 | 3.41 (.71) | .90 |
|  | Agreeableness | .28 | .55 | .36 | .07 | -.24 | -.61 | -.32 | -.03 | 3.70 (.52) | .82 |
|  | Conscientiousness | .36 | .59 | .19 | .05 | -.24 | -.45 | -.30 | .02 | 3.66 (.66) | .88 |
|  | Openness to Experience | -.23 | .18 | .44 | .63 | .18 | -.14 | -.31 | -.56 | 3.71 (.59) | .84 |
| FFM facets | N_Anxiety | .06 | -.22 | -.48 | -.33 | -.12 | .15 | .66 | .27 | 3.07 (.90) | .82 |
|  | N_Depression | .04 | -.30 | -.62 | -.44 | -.13 | .21 | .77 | .36 | 2.47 (.87) | .84 |
|  | N_Emotional Volatility | -.36 | -.43 | -.22 | -.06 | .38 | .60 | .40 | -.03 | 2.76 (.84) | .81 |
|  | E_Sociability | -.29 | .10 | .49 | .60 | .38 | .08 | -.49 | -.50 | 3.51 (.85) | .81 |
|  | E_Assertiveness | -.29 | .13 | .42 | .63 | .35 | .09 | -.45 | -.51 | 3.08 (.87) | .79 |
|  | E_Energy Level | -.26 | .26 | .61 | .57 | .39 | -.08 | -.62 | -.52 | 3.63 (.71) | .77 |
|  | A_Compassion | .18 | .43 | .19 | -.04 | -.21 | -.39 | -.12 | -.01 | 3.96 (.64) | .69 |
|  | A_Respectfulness | .39 | .47 | .25 | .01 | -.31 | -.63 | -.24 | .07 | 3.88 (.59) | .68 |
|  | A_Trust | .13 | .45 | .43 | .19 | -.07 | -.48 | -.42 | -.14 | 3.26 (.66) | .64 |
|  | C_Organization | .31 | .46 | .12 | -.02 | -.23 | -.36 | -.16 | .08 | 3.62 (.93) | .86 |
|  | C_Productiveness | .25 | .59 | .27 | .17 | -.10 | -.38 | -.43 | -.09 | 3.71 (.73) | .74 |
|  | C_Responsibility | .36 | .49 | .10 | -.01 | -.29 | -.42 | -.19 | .04 | 3.63 (.64) | .70 |
|  | O_Intellectual Curiosity | -.24 | .07 | .32 | .58 | .21 | -.06 | -.30 | -.49 | 3.80 (.65) | .68 |
|  | O_Aesthetic Sensitivity | -.13 | .16 | .28 | .32 | .02 | -.21 | -.09 | -.35 | 3.53 (.91) | .81 |
|  | O_Creative Imagination | -.20 | .20 | .48 | .68 | .24 | -.03 | -.41 | -.54 | 3.81 (.65) | .76 |

*Note*. N = Neuroticism E = Extraversion, A = Agreeableness, C = Conscientiousness, O = Openness to Experience. Correlations greater than |.12| are significant at *p* < .05 (two-tailed).

**
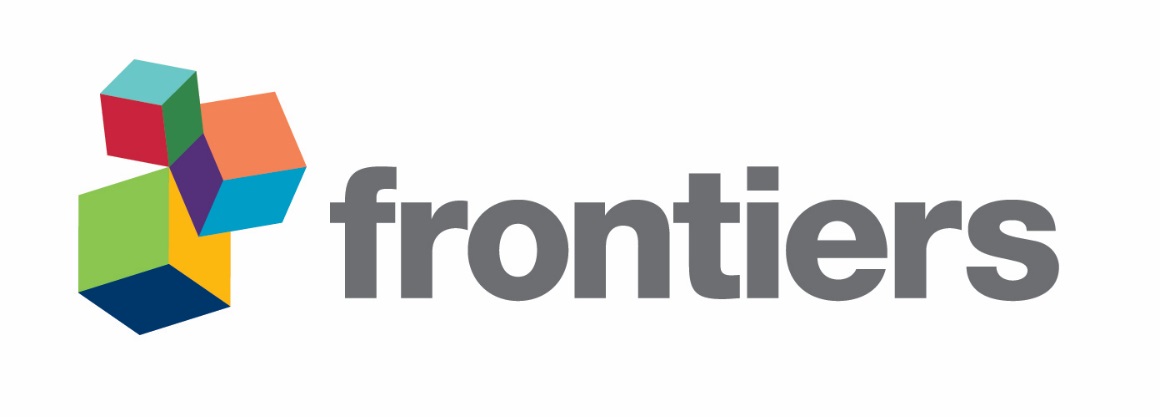
**
